# Interleukin-1α inhibitor bermekimab in patients with atopic dermatitis: randomized and nonrandomized studies

**DOI:** 10.1007/s00403-024-03319-z

**Published:** 2024-08-30

**Authors:** Eric L. Simpson, Emma Guttman-Yassky, Jeffrey Pawlikowski, Eric G. Ghorayeb, Takayuki Ota, Mark G. Lebwohl

**Affiliations:** 1 Oregon Health & Science University, South Waterfront, 3303 S. Bond Ave, Portland, OR 97239 USA; 2 Department of Dermatology, Icahn School of Medicine at Mount Sinai, 5 East 98th Street, New York, NY 10029 USA; 3 Janssen Scientific Affairs, LLC, Horsham, PA 19044 USA; 4 Immunology Global Medical Affairs, Janssen Pharmaceutical Companies of Johnson & Johnson, Horsham, PA 19044 USA; 5 Janssen Research and Development, LLC, 3210 Merryfield Row, San Diego, CA 92121 USA

**Keywords:** Atopic dermatitis, Bermekimab, MABp1, Phase 2, Subcutaneous, Interleukin-1α

## Abstract

Bermekimab is a human-derived recombinant monoclonal antibody that exhibits immunoregulatory activity by specifically blocking interleukin-1α activity. Four phase 2 studies evaluated efficacy and safety of bermekimab in patients with moderate-to-severe atopic dermatitis (AD). In addition, a novel human skin explant model was developed to assess bermekimab pharmacokinetics/pharmacodynamics and proteomic/transcriptomic effects. Study 1 (NCT03496974, *N* = 38) was an open-label, dose escalation study of subcutaneous bermekimab (200 mg or 400 mg). Study 2 (NCT04021862, *N* = 87) was a double-blind, placebo-controlled, randomized (1:1:1) study of subcutaneous bermekimab (400 mg every week (qw) or every 2 weeks) or placebo. GENESIS (NCT04791319, *N* = 198) was a double-blind, placebo- and active-comparator-controlled, randomized (1:1:2:2) study of placebo, subcutaneous bermekimab (350 mg or 700 mg qw), or dupilumab. LUNA (NCT04990440, *N* = 6) was a double-blind, placebo-controlled, randomized (4:1) study of intravenous bermekimab 800 mg qw or placebo. A novel human ex vivo skin pharmacodynamic assay supported phase 0 (NCT03953196) and phase 1 (NCT04544813) studies. In Study 1, 400 mg subcutaneous bermekimab showed improvement in efficacy assessments (e.g., ≥ 75% improvement of EASI over baseline, IGA 0/1, and worst itch); however, efficacy was not confirmed in Study 2 or GENESIS. Consequently, GENESIS and LUNA were terminated early. The novel human ex vivo skin pharmacodynamic assay demonstrated that bermekimab reduced downstream skin injury responses. Although bermekimab showed potential as an AD treatment in preclinical and early open-label trials, larger controlled studies (Study 2 and GENESIS) did not confirm those initial results.

**Supplementary Information:**

The online version contains supplementary material available at 10.1007/s00403-024-03319-z.

## Introduction

Atopic dermatitis (AD) is a chronic, heterogenous, inflammatory skin disease that adversely affects patients’ quality of life, especially in young children [1–6]. The pathophysiology of AD is complex, involving factors such as neuroinflammation, skin barrier dysfunction, and immune dysregulation [7, 8], including dysregulated interleukin (IL)-4, IL-13, and IL-31, which are cytokines associated with itch and pain [9–11]. The AD clinical phenotype is highly variable, exhibiting heterogeneity among age, ethnicity, and disease severity [2, 12].

Models of disease initiation identify keratinocyte-derived cytokines as potential initiators of AD inflammation. IL-1α is hypothesized to initiate inflammatory responses from skin barrier disruption, which is a hallmark of AD [13, 14]. As an archetypical proinflammatory cytokine, the function of IL-1α is largely mediated by the IL-1 receptor, which is present on a variety of cells, including those of the innate immune system, selective T-cell populations in the adaptive immune system, and non-immune tissues [15–17]. When activated, leukocytes (e.g., platelets, macrophages, and neutrophils) produce IL-1α, which triggers the inflammatory process [13, 14, 18] and is associated with biologic effects such as induction of matrix metalloproteinase-9 [19] and induction of inflammatory cell infiltrate in skin [20]. Since IL-1α plays a key role in the pathophysiology of many skin disorders, targeting IL-1α may provide an effective treatment to block the inflammatory process that drives a wide array of diseases, including dermatologic conditions such as AD [18, 21, 22].

Bermekimab (formerly known as MABp1) is a recombinant human immunoglobulin G1 monoclonal antibody that blocks IL-1α activity specifically. Its heavy and light chain sequences are identical to those originally expressed by a peripheral blood B lymphocyte obtained from a healthy individual. Consequently, it is considered a “true” human antibody, which is expected to be non-immunogenic in humans. Bermekimab was previously reported to be effective at reducing signs and symptoms in phase 2 studies with patients with dermatological diseases (psoriasis, acne vulgaris, and hidradenitis suppurativa) [23–25].

There are limited treatment options for patients with moderate-to-severe AD. The safety and efficacy of subcutaneous (SC) bermekimab were evaluated in patients with moderate-to-severe AD in 4 phase 2 clinical trials of increasing rigor, starting with a small open-label study with 2 dosing levels (NCT03496974; Study 1), progressing to a small, randomized, placebo-controlled study (NCT04021862; Study 2), and followed by a larger randomized, placebo- and active-comparator-controlled, phase 2b study (NCT04791319; GENESIS). In addition, intravenous (IV) bermekimab was tested in a small, randomized phase 2a study (NCT04990440; LUNA).

The results of these studies are presented in this paper, along with results from a novel human ex vivo skin pharmacodynamic (PD) assay developed to assess proteomic and transcriptomic effects associated with blockade of IL-1α-mediated, injury-induced, tissue inflammation to further support the understanding of the pharmacokinetics (PK) and PD of bermekimab.

## Materials and methods

### Study designs

Study 1 was an open-label, dose escalation study with SC bermekimab 200 mg and 400 mg every week (qw) in 2 separate cohorts of patients. Study 2 was randomized, double-blind, and placebo-controlled to evaluate SC bermekimab 400 mg qw and every 2 weeks (q2w). GENESIS was a randomized, double-blind, placebo- and active-comparator-controlled, multicenter, interventional phase 2b study with SC bermekimab 350 mg and 700 mg qw, and included a comparator/reference dupilumab [26] from week 2 through week 14. LUNA was a double-blind, randomized, placebo-controlled, multicenter, interventional phase 2a study, with an IV infusion qw of either bermekimab (800 mg in Part A; 1200 mg in Part B; ≥800 to ≤ 2400 mg in Part C) or placebo (Supplementary Methods and Figs. S1 to S4).

All human studies were approved by the respective authors’ Institutional Review Boards (IRBs), and the studies’ protocols and amendments were reviewed by an Independent Ethics Committee or IRB. The studies were performed in accordance with the Declaration of Helsinki (64th World Medical Association General Assembly, Fortaleza, Brazil, October 2013), and conducted in compliance with the International Conference on Harmonisation of Technical Requirements for Pharmaceuticals for Human Use Guidelines on Good Clinical Practice.

### Patient populations

Eligibility criteria were generally similar for all studies (Supplementary Methods). Briefly, all patients provided written informed consent to participate and were at least 18 years old, had an inadequate response to topical treatment for AD, failed to tolerate topical medications, or topical medications were medically inadvisable, and had chronic AD (≥ 3 years for Study 1 and Study 2 or ≥ 1 year for GENESIS and LUNA). Patients also had an Eczema Area and Severity Index (EASI) score ≥ 16, an IGA score ≥ 3, and ≥ 10% body surface area involvement at both screening and baseline. Exclusion criteria included prior use of topical corticosteroids or topical calcineurin inhibitors to treat AD within 7–28 days prior to baseline.

### Study objectives and endpoints

The primary objectives for Study 1 were safety and tolerability, while efficacy was a primary objective for the remaining studies, as determined by the proportion of patients with EASI-75 at week 16 (Table 1).

**Table 1:** Primary and secondary objectives/endpoints for the phase 2 studies

	Primary Objectives/Endpoints	Secondary Objectives/Endpoints
Study 1	Safety Tolerability	EASI: change from baseline to visit 8; % achieving ≥ 50% reduction IGA: % patients achieving IGA (0/1) at visit 8; % patients achieving ≥ 2 IGA score reduction at visit 8 PK assessment NRS: % change for peak weekly averaged pruritus and pain NRS from baseline to visit 8; change in weekly averaged peak NRS from baseline to visit 8 DLQI: change from baseline to visit 8 GISS: change from baseline to visit 8 HADS: change from baseline to visit 8 POEM: % change from baseline to visit 8 SCORAD: change in SCORAD score from baseline to visit 8; % achieving ≥ 50% reduction from baseline at visit 8 Visit 1 Questionnaire for pruritus, pain, and erythema: % change from pre- and post-injection
Study 2	Efficacy: % patients achieving EASI-75 at week 16 Safety Tolerability	Evaluation of AD symptoms at weeks 4, 8, 12, 16, and 32: % patients achieving ≥ 4-point improvement in weekly average peak daily and overall pruritus and pain NRS score from baseline; % patients achieving IGA score of 0, 0/1, or ≤ 2 in combination with a reduction in this measure from baseline ≥ 2 points at week 16; % achieving EASI-90; changes in DLQI, HADS (Anxiety, Depression), POEM, SCORAD, and GISS from baseline PK assessment Immunogenicity – antibodies to bermekimab
GENESIS	Efficacy: EASI-75 at week 16	At week 16: Proportion of patients with: vIGA-AD of 0/1 and ≥ 2-point reduction from baseline; improvement of eczema-related itch NRS ≥ 4 from baseline to week 16 among patients with a baseline itch value ≥ 4; proportion of patients with EASI-90, EASI-75 Safety and tolerability: number of TEAEs, SAEs Immunogenicity: antibodies to bermekimab
LUNA	Efficacy of 16 weeks of multiple IV doses of bermekimab compared to placebo: EASI-75 at week 16	PK Immunogenicity Safety and tolerability at 16 weeks

AD, atopic dermatitis; AE, adverse event; DLQI, Dermatology Life Quality Index; EASI, Eczema Area and Severity Index; EASI-75, 75% improvement of EASI from baseline; EASI-90, 90% improvement of EASI from baseline; GISS, Global Individual Sign Score; HADS, Hospital Anxiety and Depression Scale; IGA, Investigator Global Assessment; IV, intravenous; NRS, Numeric Rating Scale; PK, pharmacokinetics; POEM, Patient Oriented Eczema Measure; SAE, serious adverse event; SCORAD, Severity scoring Atopic Dermatitis; TEAE: Treatment-Emergent Adverse Event; vIGA-AD, validated IGA for AD.

### Skin biopsies for ex vivo pharmacodynamic skin assay

A skin injury assay was developed using 4 mm skin biopsies from 10 healthy donors in a phase 0 study [27] (Supplementary Methods and Fig. S5). Ex vivo blockage of IL-1α with this assay can result in the attenuation of injury-induced inflammation, which can be monitored both at a proteomic and transcriptomic level (Fig. S6).

### Statistical analysis

Descriptive statistics, including mean, median, standard deviation (SD), minimum, and maximum, were used to summarize continuous variables for Study 1, Study 2, and GENESIS. Counts and percentages were used to summarize categorical variables, and graphical data displays and patient listings were used to summarize the data (see additional statistical analysis details in the Supplementary Methods). Due to early termination of LUNA, no formal analyses were performed.

## Results

### Patient characteristics

Overall, patient demographics and baseline AD disease characteristics were similar for Study 1, Study 2, and GENESIS (as shown in Tables 2 and 3), and also with LUNA, which involved patients ranging from 19 to 51 years, a body mass index range of 17.3 to 37.9 kg/m^2^, and an AD disease duration ranging from 19.0 to 41.6 years. Five of the LUNA patients were Hispanic or Latino and were predominantly White (*n* = 4) with 1 American Indian or Alaska Native patient and 1 Black or African American patient. Treatment disposition through week 16 for all 4 studies is summarized in Table 4.

**Table 2:** Patient demographics for Study 1, Study 2, and GENESIS

	Study 1 Bermekimab 200 mg qw	Study 1 Bermekimab 400 mg qw	Study 2 Placebo	Study 2 Bermekimab 400 mg qw	Study 2 Bermekimab 400 mg q2w	GENESIS Placebo	GENESIS Bermekimab 350 mg qw	GENESIS Bermekimab 700 mg qw	GENESIS Dupilumab 300 mg q2w
*N*	10	28	29	29	29	33	33	67	65
Age, mean, yrs (SD)	46.2 (16.78)	48.5 (19.1)	50.7 (15.59)	41.9 (16.65)	44.0 (15.71)	35.1 (14.18)	34.6 (13.32)	36.0 (12.30)	37.2 (15.02)
Gender									
Male, *n*, (%)	3 (30)	10 (35.7)	9 (31.0)	9 (31.0)	11 (37.9)	20 (60.6)	19 (57.6)	39 (58.2)	33 (50.8)
Race, *n* (%)									
Asian	1 (10)^a^	0^a^	0	1 (3.4)	2 (6.9)	6 (18.2)	7 (21.2)	14 (20.9)	10 (15.4)
Black or African American	4 (40)	2 (7.1)	6 (20.7)	8 (27.6)	8 (27.6)	2 (6.1)	1 (3.0)	2 (3.0)	7 (10.8)
Native Hawaiian or Other Pacific Islander	-	-	-	-	-	0	1 (3.0)	0	0
White	5 (50)	26 (92.9)	23 (79.3)	20 (69.0)	19 (65.5)	25 (75.8)	20 (60.6)	51 (76.1)	46 (70.8)
Other	-	-	0	0	0	-	-	-	-
Multiple	-	-	-	-	-	0	2 (6.1)	0	0
Not reported	-	-	-	-	-	0	2 (6.1)	0	1 (1.5)
Unknown	-	-	-	-	-	0	0	0	1 (1.5)
Ethnicity									
Hispanic or Latino	-	-	18 (62.1)	15 (51.7)	14 (48.3)	0	0	5 (7.5)	4 (6.2)
Not Hispanic or Latino	-	-	11 (37.9)	14 (48.3)	15 (51.7)	33 (100.0)	31 (93.9)	62 (92.5)	59 (90.8)
Not reported	-	-	-	-	-	0	2 (6.1)	0	2 (3.1)
Median Weight, kg	78.5	67.8	77.0	82.0	82.0	80.0	74.5	74.0	74.7
Median BMI, kg/m^2^	NA	NA	27.2	29.0	30.3	24.8	24.9	25.5	25.4

^a^Category listed as “Asian, White” for Study 1

Note: N’s for each parameter reflect non-missing values

BMI, body mass index; N, number of patients; NA, not applicable; qw, every week; q2w, every 2 weeks; SD, standard deviation; yrs, years

**Table 3:** Patient baseline disease characteristics for Study 1, Study 2, and GENESIS

	Study 1 Bermekimab 200 mg qw	Study 1 Bermekimab 400 mg qw	Study 2 Placebo	Study 2 Bermekimab 400 mg qw	Study 2 Bermekimab 400 mg q2w	GENESIS Placebo	GENESIS Bermekimab 350 mg qw	GENESIS Bermekimab 700 mg qw	GENESIS Dupilumab 300 mg q2w
*N*	10	28	29	29	29	33	33	67	65
Median disease duration, yrs	NA	NA	9.7	14.0	10.0	21.0	25.0	24.0	21.0
Median BSA	NA	NA	28.8	33.3	31.8	42.0	41.0	45.0	45.0
Median EASI	25.6	28.4	21.5	20.7	19.8	27.6	26.6	26.1	25.7
IGA, *n* (%)									
Clear (0)	NA	NA	0	0	0	0	0	0	0
Almost clear (1)	NA	NA	0	1 (3.4)	0	0	0	0	0
Mild (2)	NA	NA	0	0	2 (6.9)	0	0	0	0
Moderate (3)	NA	NA	20 (69.0)	17 (58.6)	17 (58.6)	22 (66.7)	19 (57.6)	39 (58.2)	39 (60.0)
Severe (4)	NA	NA	9 (31.0)	11 (37.9)	10 (34.5)	11 (33.3)	14 (42.4)	28 (41.8)	26 (40.0)
Median SCORAD	61.8	69.7	61.2	61.3	61.6	60.2	64.4	65.4	65.4
Median POEM	18.5	16.5	16.0	17.0	16.0	20.0	23.0	22.0	20.0
Median DLQI	10	10.5	15.0	13.0	10.0	12.0	14.0	17.0	16.0
Median NRS overall itch	8	8	8.3^a^	7.8^a^	7.5^a^	6.6^a^	7.2^a^	7.4^a^	7.4^a^
Median NRS overall pain	NA	6.1	4.6^b^	6.0^b^	5.0^b^	5.5^b^	6.0^b^	6.7^b^	6.8^b^
Biologic therapy for AD, *n* (%)	NA	NA	1 (3.4)	3 (10.3%)	1 (3.4)	0	0	0	0

^a^NRS weekly average of worst daily skin itch

^b^NRS weekly average of worst daily skin pain

AD, atopic dermatitis; BSA, body surface area; DLQI, Dermatology Life Quality Index (10-item questionnaire, 0 [not at all] to 3 [very much]); EASI, Eczema Area and Severity Index (4 AD disease characteristics: 0 [absent] through 3 [severe]; area of involvement score: 0 to 6); IGA, Investigator Global Assessment (5-point scale: 0 [clear] to 4 [severe]); N, number of patients; NA, not available; NRS, Numerical Rating Scale (itch intensity: 0 – “no itch” to 10 – “worst itch imaginable”; pain intensity: 0 – “no pain” to 10 – “most severe pain”); POEM, Patient Oriented Eczema Measure (7-item questionnaire, 0 = no days, 1 = 1 to 2 days, 2 = 3 to 4 days, 3 = 5 to 6 days, 4 = every day); qw, every week; q2w, every 2 weeks; SCORAD, Severity Scoring of Atopic Dermatitis (A = extent of affected body surface area, B = severity, C = subjective symptoms; A/5 + 7B/2 + C, with maximum of 103); SD, standard deviation; yrs, years

**Table 4:** Treatment disposition through week 16

	Study 1	Study 1	Study 2	Study 2	Study 2	GENESIS	GENESIS	GENESIS	GENESIS	LUNA
	**Berm 200 mg** **qw**	**Berm 400 mg** **qw**	**Placebo**	**Berm 400 mg** **qw**	**Berm 400 mg q2w**	**Placebo**	**Berm 350 mg** **qw**	**Berm 700 mg** **qw**	**Dupilumab 300 mg** **q2w**	**Berm 800 mg** **qw**
Full Analysis Set, *N*	10	28	29	29	29	33	33	67	65	4
Discontinued study, *n* (%)	10 (100)^a^	28 (100)^a^	2 (6.9)	2 (6.9)	1 (3.4)	14 (42.4)	12 (36.4)	37 (55.2)	27 (41.5)	4 (100)
Reason for discontinuation, *n* (%)										
Adverse event	-	2 (7.1)	2 (6.9)	0	0	1 (3.0)	0	5 (7.5)	0	-
Worsening of AD	-	-	-	-	-	1 (3.0)	0	4 (6.0)	0	-
Other AE	-	-	-	-	-	0	0	1 (1.5)	0	-
Death	-	-	0	0	0	0	0	0	0	-
Lack of efficacy	-	-	-	-	-	1 (3.0)	1 (3.0)	4 (6.0)	1 (1.5)	-
Lost to follow-up	-	1 (3.6)^b^	-		-	1 (3.0)	0	0	1 (1.5)	-
Non-compliance with study drug	-	-	-	-	-	0	0	0	0	-
Pregnancy	-	-	0	1 (3.4)	0	0	0	0	0	-
Protocol violation	-	-	-	-	-	0	0	1 (1.5)	0	-
Site terminated by Sponsor	-	-	-	-	-	0	0	1 (1.5)	0	-
Study terminated by Sponsor	-	-	0	0	0	9 (27.3)	8 (24.2)	20 (29.9)	21 (32.3)	4 (100)
Removed from study by PI	-	1 (3.6)	-	-	-	-	-	-	-	-
Withdrawal by patient	1 (10)	4 (14.3)	0	0	1 (3.4)	1 (3.0)	2 (6.1)	3 (4.5)	2 (3.1)	-
Patient completed study	9 (90)	20 (71.4)	-	-	-	-	-	-	-	-
Other	-	-	0	1 (3.4)	0	1 (3.0)	1 (3.0)	3 (4.5)	2 (3.1)	-

^a^Percentages are based on all enrolled patients

^b^After 3 unsuccessful contact attempts. AD, atopic dermatitis; AE, adverse event; Berm, bermekimab; PI, Principal Investigator; qw, every week; q2w, every 2 weeks

The analysis for Study 1 included a total of 38 patients in the intent-to-treat population who received ≥ 1 bermekimab dose. Concomitant topical corticosteroids and topical calcineurin inhibitors were not allowed during the study. Of the 87 patients in Study 2 who were randomized and dosed, each group (placebo, bermekimab 400 mg qw, and bermekimab 400 mg q2w) consisted of 29 patients. In GENESIS, 198 patients were randomized and received ≥ 1 dose: 33 patients in placebo group, 33 patients in the bermekimab 350 mg group, 67 patients in the bermekimab 700 mg group, and 65 patients in the dupilumab group. One patient was randomized to the dupilumab treatment group but was not treated. Of the 6 patients randomized in LUNA, 5 were in Part A (4 patients to the bermekimab 800 mg treatment arm and 1 patient to the placebo arm), and 1 in Part B to the bermekimab 1200 mg treatment arm due to an interactive web response system error.

### Efficacy

**Fig. 1:**
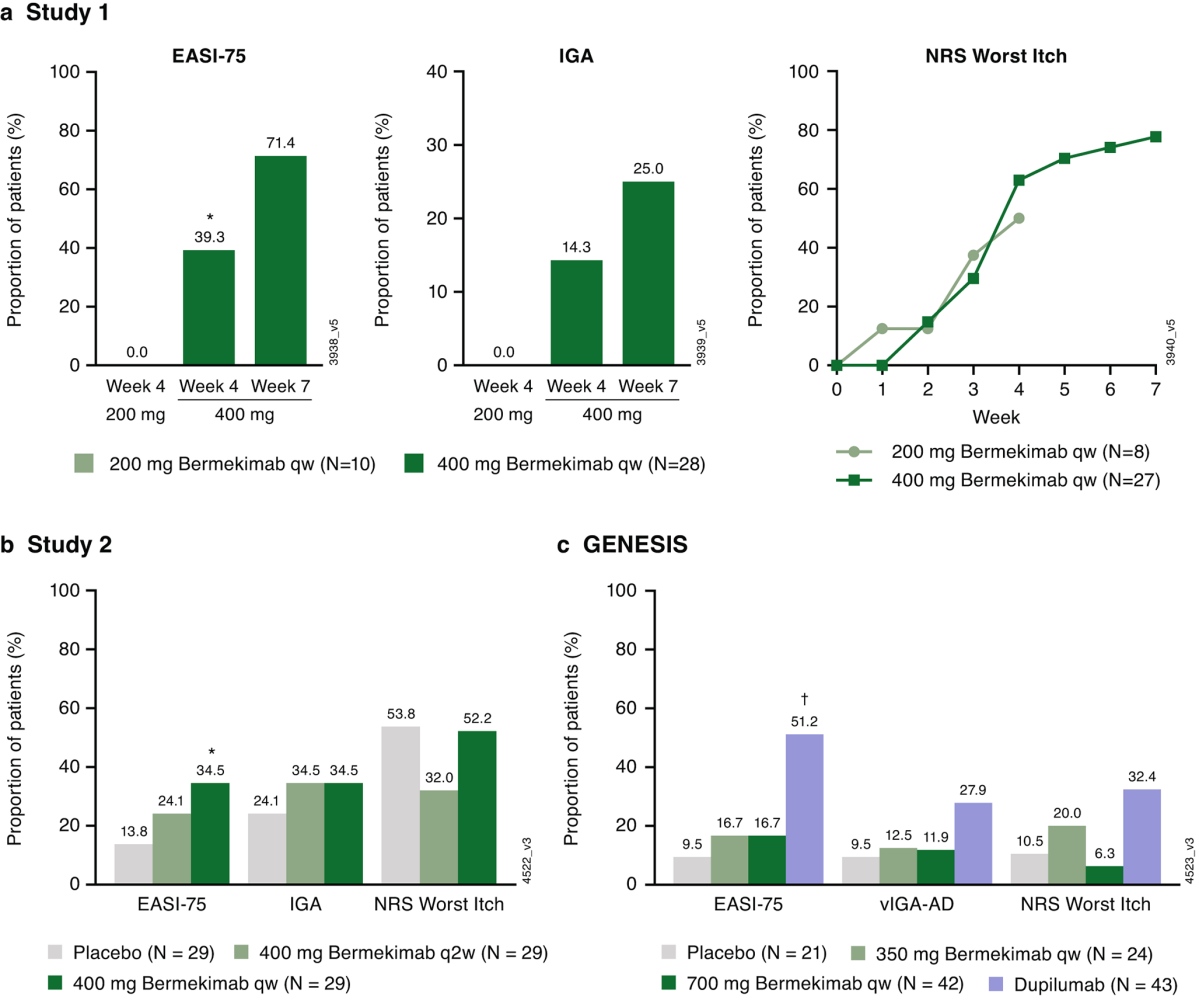
Clinical efficacy of subcutaneous bermekimab. **(a)** Proportions of patients in Study 1 who achieved EASI-75, IGA score of 0 or 1 and ≥ 2 point reduction from baseline, or ≥ 4-point reduction from baseline in NRS worst daily itch score among patients with baseline NRS ≥ 4 through week 7. Missing data were imputed using last observation carried forward. **(b)** Proportions of patients in Study 2 who achieved EASI-75, IGA ≤ 2 and ≥ 2-point reduction from baseline, or ≥ 4-point reduction from baseline in NRS worst daily itch score among patients with baseline NRS ≥ 4 at week 16. Patients with missing data or who discontinued study treatment were assumed to be nonresponders. **(c)** Proportions of patients in GENESIS who achieved EASI-75, vIGA-AD score of 0 or 1 and ≥ 2-point reduction from baseline, and ≥ 4 reduction in NRS worst daily itch score among patients with baseline NRS ≥ 4 at week 16. *P = 0.04 versus bermekimab 200 mg qw for Panel a and versus placebo for Panel b, †*P* = 0.001 versus placebo. EASI-75, 75% improvement from baseline in Eczema and Severity Index; IGA, Investigator Global Assessment; NRS, Numerical Rating Scale; qw, every week; q2w, every 2 weeks; vIGA-AD, validated Investigator Global Assessment – Atopic Dermatitis

In Study 1, all primary and secondary efficacy analyses were performed for the safety analysis set, which consisted of all patients who received ≥ 1 dose of study medication. Clinically and statistically significant improvement was observed for all efficacy endpoints in the highdose group, as presented at the American Academy of Dermatology 2019 Annual Meeting [26]. Additionally, a significant dose response for the high-dose group compared to the low-dose group was observed for key endpoints. Efficacy analysis of the endpoints at week 7 for the 400 mg group demonstrated that mean changes from baseline for all outcome measures were statistically significant (*P* < 0.001) (data not shown). In addition, at week 7, 71.4% of patients in the 400 mg group achieved 75% improvement from baseline in Eczema and Severity Index (EASI-75) and 25% had an Investigator Global Assessment (IGA) score of 0/1 and ≥ 2-point reduction from baseline (Fig. 1a). An increased proportion of patients in the 400 mg group showing improved AD symptoms was also observed over time, with a ≥ 4-point reduction in Numerical Rating Scale (NRS) worst daily itch among patients with baseline NRS ≥ 4.

In Study 2, the full analysis set, which included all patients who were randomized and received  ≥ 1 dose of study intervention, was used for efficacy analyses of the endpoints. At week 16, a significantly greater proportion of patients receiving bermekimab 400 mg qw (34.5%; 10/29 patients) achieved EASI-75 compared with those receiving placebo (13.8%; 4/29 patients; *P* = 0.04); the proportion of patients receiving bermekimab 400 mg q2w who achieved EASI-75 (24.1%; 7/29 patients) was not significantly different than for those receiving placebo (*P* = 0.35) (Fig. 1b). Results appeared consistent across EASI components (data not shown). The proportion of patients with an IGA score of mild or better (≤ 2) and at least 2-grade improvement was 34.5% (10/29; *P* = 0.35) and 34.5% (10/29; *P* = 0.42) for 400 mg bermekimab qw and q2w, respectively, compared to placebo (24.1%; 7/29) at week 16. Although the response rate was numerically higher in both bermekimab arms compared to placebo, the differences were not statistically significant. The proportion of patients who had ≥ 4point improvement in NRS worst daily itch score, among those with baseline NRS ≥ 4, was 52.2% (12/23) and 32.0% (8/25) at week 16 for bermekimab 400 mg qw and 400 mg q2w, respectively, with no significant difference from placebo (53.8%; 14/26; *P* = 0.99 and *P* = 0.09, respectively).

The GENESIS interim analysis took place when approximately 50% of randomized patients who received at least 1 administration of study agent completed their week 16 visit or terminated their study participation before week 16. The criterion for futility is that the posterior probability of the bermekimab group being better than the placebo group is < 80% in EASI-75 at week 16. The futility criterion was met, and the Interim Analysis Committee recommended to consider termination of the trial. Consequently, the study was terminated early, and only 1 database lock occurred on 19Apr2022 instead of having 2 as originally planned.

The modified full analysis set was used for endpoint analyses and was defined as the randomized patients who received ≥ 1 dose of study intervention who could have reached a visit by the time the decision was made to terminate the GENESIS study on 02Feb2022 (*n* = 130: 21 placebo, 24 bermekimab 350 mg, 42 bermekimab 700 mg, and 43 dupilumab group). Compared to placebo (9.5%; 2/21 patients), the proportion of treated patients achieving EASI-75 at week 16 was 16.7% (4/24; *P* = 0.49) and 16.7% (7/42; *P* = 0.45) for 350 mg and 700 mg bermekimab, respectively, and 51.2% (22/43; *P* = 0.001) for dupilumab (Fig. 1c). Although there was some improvement observed in the symptom-based efficacy assessments for bermekimab, it was not significant compared to placebo; however, the improvement with dupilumab was significantly greater than with placebo. At week 16, there was no significant difference between the proportion of patients with a validated IGA 0/1 and ≥ 2 grade improvement in the placebo (9.5%; 2/21) and bermekimab 350 mg and 700 mg treatment groups (12.5%, 3/24, *P* = 0.76 and 11.9%, 5/42, *P* = 0.78, respectively), using the modified full analysis set; the proportion of patients in the dupilumab group was 27.9% (12/43, *P* = 0.10). Among patients with baseline NRS ≥ 4, the proportion of patients with improvement of eczema-related worst itch NRS ≥ 4 at week 16 from baseline was 10.5% (2/21) for the placebo group, 20.0% (4/24) for the bermekimab 350 mg group, 6.3% (2/42) for the bermekimab 700 mg group, and 32.4% (11/43) for the dupilumab group.

The lack of supportive positive data on efficacy in GENESIS (including EASI-75, IGA, and NRS worst itch results), particularly from the 700 mg dose group, suggested that there may not have been substantial additional benefit for patients with AD from the higher dose. Through week 16, 45.5% (90/198) of patients discontinued study treatment with the majority terminating early at the Sponsor’s discretion (29.3% [58/198] patients) followed by withdrawal of consent by patient to continue in the study (4.0% [8/198] patients).

In LUNA, the efficacy analyses were based on the full analysis set that included all 6 patients who were randomized at week 0 and received ≥ 1 dose of study intervention. Due to the small sample size and short duration of treatment, the efficacy and safety assessment was limited. Although some patients showed improvements in EASI-75 assessments at certain timepoints (data not shown), no meaningful conclusions could be drawn from these results.

### Safety

Overall, bermekimab was well tolerated in the 4 phase 2 studies. No new safety concerns were identified, and no deaths were reported.

In Study 1, 18 patients reported non-serious adverse events (AEs), and most (72%) AEs were grade 1 (Table S1). Only 1 grade 3 AE was reported, which was assessed as an exacerbation of the patient’s pre-existing hypertension possibly related to study drug but resolved without a change in study treatment. Remaining AEs were mild to moderate in severity. There were no serious AEs (SAEs) reported.

In Study 2, the proportion of patients experiencing at least 1 AE through week 16 was 34.5% (10/29) and 37.9% (11/29) in the bermekimab 400 mg qw and bermekimab 400 mg q2w groups, respectively, compared with 24.1% (7/29) in the placebo group (Table 5). Three, 0, and 2 patients in the bermekimab 400 mg qw, bermekimab 400 mg q2w, and placebo groups, respectively, were reported to have experienced AEs leading to discontinuation through week 16. One SAE was reported in the bermekimab 400 mg qw group, and none were reported in the bermekimab 400 mg q2w or placebo groups. Following crossover to bermekimab at Week 16, the number of patients experiencing at least 1 AE in the placebo to bermekimab 400 mg qw group through Week 36 was 4 (14.8%). One patient (3.4%) in the bermekimab 400 mg qw group, who had a pre-existing medical history of cellulitis with hospitalization, was reported to have experienced 1 episode of worsening AD and 2 SAEs of cellulitis which required hospitalization but were considered by the Investigator not to be related to the study agent.

**Table 5:** Summary of treatment-emergent adverse events with frequency of ≥ 5 for Study 2 and GENESIS

	Study 2 Placebo	Study 2 Bermekimab 400 mg qw	Study 2 Bermekimab 400 mg q2w		GENESIS Placebo	GENESIS Bermekimab 350 mg qw	GENESIS Bermekimab 700 mg qw	GENESIS Dupilumab 300 mg q2w
Avg duration of follow-up (weeks)	15.40	15.50	16.27		14.47	14.18	13.89	14.92
Avg exposure (number of administrations)	15.21	15.03	15.66		12.21	14.18	13.89	14.92
Patients with ≥ 1 AE, *n* (%)	7 (24.1)	10 (34.5)	11 (37.9)		18 (54.5)	23 (69.7)	43 (64.2)	33 (50.8)
System Organ Class								
Preferred Term								
Infections and infestations								
Influenza	0	0	2 (6.9)		-	-	-	-
Nasopharyngitis	2 (6.9)	0	1 (3.4)		2 (6.1)	3 (9.1)	8 (11.9)	6 (9.2)
Upper respiratory tract infection	0	1 (3.4)	0		1 (3.0)	3 (9.1)	4 (6.0)	4 (6.2)
COVID-19	-	-	-		4 (12.1)	2 (6.1)	1 (1.5)	2 (3.1)
Skin and subcutaneous tissue disorders								
Dermatitis atopic	1 (3.4)	1 (3.4)	0		4 (12.1)	4 (12.1)	11 (16.4)	4 (6.2)
Respiratory, thoracic and mediastinal disorders								
Asthma	0	2 (6.9)	1 (3.4)		-	-	-	-
Rhinorrhea	1 (3.4)	0	0		0	2 (6.1)	0	0
Nervous system disorders								
Headache	0	0	1 (3.4)		0	0	5 (7.5)	5 (7.7)
Musculoskeletal and connective tissue disorders								
Back pain	-	-	-		2 (6.1)	1 (3.0)	2 (3.0)	0
General disorders and administration site conditions								
Fatigue	0	0	2 (6.9)		-	-	-	-
Injection site swelling	0	0	2 (6.9)		-	-	-	-
Injection site erythema	0	0	1 (3.4)		0	2 (6.1)	5 (7.5)	0
Metabolism and nutrition disorders								
Decreased appetite	0	0	3 (10.3)		-	-	-	-
Gastrointestinal disorders								
Constipation	0	0	2 (6.9)		-	-	-	-

AE, adverse event; avg, average; COVID-19, novel coronavirus disease 2019; qw, every week; q2w, every 2 weeks

In GENESIS, 198 patients in the study (54.5% [18/33] of patients in the placebo group, 66.0% [66/100] of patients in the combined bermekimab treatment group, and 50.8% [33/65] of patients in the dupilumab treatment group) reported ≥ 1 AEs through week 16. The most commonly reported AEs were AD, nasopharyngitis, and upper respiratory tract infection (Table 5). No SAEs were reported in the placebo and dupilumab treatment groups. SAEs reported in the bermekimab-treated groups were 2 events of worsening AD (1 each in the 350 mg and 700 mg treatment groups), and 1 each of severe aspartate aminotransferase increased and auricular hematoma (700 mg group). However, none of these SAEs were assessed by the Investigator as related to the study intervention. Of note, the SAE of aspartate aminotransferase increased occurred on Study Day 1 prior to study intervention administration. No new SAEs were reported after week 16 through week 36. Through week 16, AD was the primary reason for 1 patient from the placebo group and 4 patients from the bermekimab 400 mg group to discontinue the study, and 1 patient from the bermekimab 400 mg group discontinued due to moderate folliculitis. No discontinuations were reported in the dupilumab group.

In LUNA, 5 patients reported a total of 7 AEs, which were assessed as mild or moderate in intensity; all but 2 of the AEs resolved. There were 3 cases of worsening AD reported (2 patients who received bermekimab 800 mg and 1 patient who received placebo), and 1 case each of otitis externa, hypokalemia, and vulvovaginal candidiasis. No safety concerns were reported for the patient who received the accidental overdose after mistakenly being assigned to a higher dose cohort (bermekimab 1200 mg).

### Ex vivo skin pharmacodynamic skin assay

To further support the understanding of the PK/PD of bermekimab, a novel phase 0 ex vivo skin PD assay was developed to monitor proteomic and transcriptomic changes associated with blockade of the IL-1α-mediated, injury-induced inflammatory response [27]. Inflammatory cytokine and chemokine proteins were upregulated with 24 h of ex vivo culture compared to control before 800 mg SC bermekimab treatment (Fig. S7). In the phase 1 ex vivo bermekimab PD assay, SC bermekimab 800 mg induced greater suppression of IL-6 and IL-8 protein expression compared to SC bermekimab 200 mg and 400 mg (Fig. S8).

## Discussion

Data from earlier phase clinical studies in hidradentitis suppurativa [25] and psoriasis [23] using bermekimab revealed significant therapeutic activity with the IL-1⍺ target, and bermekimab monoclonal antibody therapy appeared to be effective at specifically targeting IL-1α in addition to reducing the severity of AD, including rapid reduction in itch and pain. The relevance of IL-1α as a key skin alarmin driving tissue injury inflammation was supported by the phase 1 ex vivo PD data from our novel human ex vivo skin PD assay, which demonstrated that bermekimab reduced downstream skin injury responses, with maximal inhibition observed at bermekimab doses > 400 mg. SC bermekimab was also demonstrated to downregulate inflammatory proteins in ex vivo skin explants, which provided proof of concept that IL-1α is decreased.

Since there still are limited treatment options for moderate-to-severe AD that act across the ethnically/racially-diverse AD patient spectrum [6, 12, 28–30], the potential of bermekimab as a novel therapeutic agent was further investigated in randomized, placebo-controlled phase 2 and 2b studies following results from an initial small open-label phase 2 study that demonstrated efficacy of bermekimab to reduce the severity of AD while rapidly decreasing itch. Bermekimab was well tolerated at doses ranging from 200 mg to 700 mg in the phase 2 trials described in this paper; however, although bermekimab 400 mg qw was superior to placebo for the primary endpoint of EASI-75 at week 16 in Study 2, other efficacy endpoints, including additional assessments of skin inflammation and itch, did not show consistent benefit of bermekimab 400 mg qw or q2w compared with placebo. In addition, results of the more rigorous phase 2b GENESIS study did not demonstrate sufficient efficacy at reducing the severity of AD, including reduction of itch. No clear difference was observed between bermekimab doses of 350 mg and 700 mg, while dupilumab results were consistent with previously published results [31]. There was some improvement in the symptoms of the bermekimab-treated groups based on the efficacy assessment but those were not significant compared to the dupilumab arm. Consequently, the lack of supportive data on efficacy, particularly from the 700 mg cohort in the GENESIS study, suggests that there may not be substantial additional benefit for AD patients from a higher bermekimab dose.

Epithelium-derived cytokines, such as IL-33, IL-25, and thymic stromal lymphopoietin are alarmins that are involved with type-2, type-1, and type-17 immune responses, and are associated with autoimmune diseases and allergic disorders [32, 33]. Ongoing research about the use of biologics to target alarmins suggests that alarmins might have potential to treat allergy, asthma, and possibly cancer and autoimmune diseases such as AD [33]. However, a lack of observed efficacy for some antibodies could be related to insufficient levels of the cytokines or limited antibody access, or perhaps these cytokines are only important for AD initiation and are not involved in AD pathology maintenance.

Limitations of the phase 2 studies described in this paper include the relatively small sample sizes, as well as the heterogeneity of the AD population; therefore, results should be interpreted with caution. Study 1 was open-label and not placebo-controlled, which limits making conclusions about efficacy. In the GENESIS study, a small number of patients who had NRS ≥ 4 at baseline may have impacted the analysis of the proportion of patients with eczema-related itch NRS ≥ 4.

In conclusion, SC bermekimab was well tolerated in patients with AD, and no new safety findings were observed. Although bermekimab initially appeared to have potential as a novel and effective treatment for AD in one early phase 2 clinical trial, the more rigorously designed trial, the GENESIS phase 2b study, did not confirm those results, suggesting that blocking IL-1α with bermekimab does not provide a viable approach to AD monotherapy.

## Electronic supplementary material

Below is the link to the electronic supplementary material.

Supplementary Material 1

## Data Availability

The data sharing policy of Janssen Pharmaceutical Companies of Johnson & Johnson is available at https://www.janssen.com/clinical-trials/transparency. As noted on this site, requests for access to the study data can be submitted through the Yale Open Data Access (YODA) Project site at http://yoda.yale.edu.
