# Supplementary material for: Interleukin-1α inhibitor bermekimab in patients with atopic dermatitis: randomized and nonrandomized studies
Source: Arch Dermatol Res. 2024 Aug 30;316(8):589. doi: 10.1007/s00403-024-03319-z (PMC11364570; doi:10.1007/s00403-024-03319-z)
Supplement: Supplementary file 1 — Supplementary Material 1 [file 403_2024_3319_MOESM1_ESM.docx]

# Title: Interleukin-1α inhibitor bermekimab in patients with atopic dermatitis: randomized and nonrandomized studies

## Short Title: Bermekimab in Patients With Atopic Dermatitis

## Authors:

Eric L. Simpson, MD^1^, Emma Guttman-Yassky, MD^2^, PhD, Jeffrey Pawlikowski, PhD^3^, Eric G. Ghorayeb, MD^4^, Takayuki Ota, MD^5^, Mark G. Lebwohl, MD^2^

## Affiliations:

^1^Oregon Health & Science University, South Waterfront, 3303 S. Bond Ave., Portland, Oregon, 97239, USA ([simpsone@ohsu.edu](mailto:simpsone@ohsu.edu), 0000-0002-1802-7311)

^2^Department of Dermatology, Icahn School of Medicine at Mount Sinai, 5 East 98^th^ Street, New York, New York, 10029, USA ([emma.guttman@mountsinai.org](mailto:emma.guttman@mountsinai.org), 0000-0002-9363-324X; [Lebwohl@aol.com](mailto:Lebwohl@aol.com), 000-0002-4705-5303)

^3^Janssen Scientific Affairs, LLC, Horsham, Pennsylvania, 19044, USA ([jpawliko@its.jnj.com](mailto:jpawliko@its.jnj.com); 0000-0001-6641-1038)

^4^Immunology Global Medical Affairs, Janssen Pharmaceutical Companies of Johnson & Johnson, Horsham, Pennsylvania, 19044, USA ([eghoraye@its.jnj.com](mailto:eghoraye@its.jnj.com), 0009-0004-0675-330X)

^5^Janssen Research and Development, LLC, 3210 Merryfield Row, San Diego, California, 92121, USA ([tota3@its.jnj.com](mailto:tota3@its.jnj.com), 0000-0001-6249-0664)

## Corresponding author:

Dr. Eric Simpson

Oregon Health & Science University

South Waterfront, 3303 S. Bond Ave.

Portland, Oregon, 97239, USA

Email address: [simpsone@ohsu.edu](mailto:simpsone@ohsu.edu)

Phone number: 503-494-3968

# Supplementary material

**Interleukin-1α inhibitor bermekimab in patients with atopic dermatitis: randomized and nonrandomized studies**

Eric Simpson,^1^ Emma Guttman-Yassky,^2^ Jeffrey Pawlikowski,^3^ Eric Ghorayeb,^4^ Takayuki Ota,^5^ Mark G. Lebwohl^2^

^1^Oregon Health & Science University, South Waterfront, 3303 S. Bond Ave., Portland, Oregon, 97239, USA (simpsone@ohsu.edu)

^2^Department of Dermatology, Icahn School of Medicine at Mount Sinai, 5 East 98^th^ Street, New York, New York, 10029, USA (emma.guttman@mountsinai.org; Lebwohl@aol.com)

^3^Janssen Scientific Affairs, LLC, Horsham, Pennsylvania, USA (jpawliko@its.jnj.com)

^4^Immunology Global Medical Affairs, Janssen Pharmaceutical Companies of Johnson & Johnson, Horsham, Pennsylvania, USA (eghoraye@its.jnj.com)

^5^Janssen Research and Development, LLC, 3210 Merryfield Row, San Diego, California, 92121, USA (tota3@its.jnj.com)

**Corresponding author:**

Dr. Eric Simpson

Oregon Health & Science University, South Waterfront

3303 S. Bond Ave.

Portland, Oregon, 97239, USA

Email address: simpsone@ohsu.edu

Phone number: 503-494-3968

**List of institutional review boards and ethics committees**

**Study 2 (NCT04021862)**

Western Institutional Review Board 1019 39th Avenue SE, Suite 120, Puyallup, WA, 98374, US for all sites.

**GENESIS (NCT04791319)**

| **Site** | **Institutional Review Board/Ethical Committee** |
| --- | --- |
| Lynderm Research Inc.  25 Main Street Markham North  Markham, ON, L3P 1X3 | Advarra  372 Hollandview Trail, Suite 300  Aurora, ON, L4G 0A5 |
| Innovaderm Research Inc.  3530 boulevard Saint-Laurent , Suite 400  Montreal, Quebec, H2H2B5 | Advarra  372 Hollandview Trail, Suite 300  Aurora, ON, L4G 0A5 |
| Centre De Recherche Dermatologique Du Quebec Metropolitan  2880 Chemin Quatre-Bouregois, Unit 105  Quebec, QC, G1V 4X7 | Advarra  372 Hollandview Trail, Suite 300  Aurora, ON, L4G 0A5 |
| DermEdge Research  755 Queensway East, Suite 107  Mississauga, ON, L4Y 4C5 | Advarra  372 Hollandview Trail, Suite 300  Aurora, ON, L4G 0A5 |
| Dermatology Research Institute Inc.  8500 Blackfoot Trail SE, Suite 310  Calgary, AB, T2J 7E1 | Health Research Ethics Board of Alberta Clinical Trials Committee  1500  10104 - 103 Avenue NW  Edmonton, Alberta, T5J 0H8 |
| Allergy Research Canada Inc.  8279 Lundy’s Lane, Unit A3-A4  Niagara Falls, Ontario, L2H 1H5 | Advarra  372 Hollandview Trail, Suite 300  Aurora, ON, L4G 0A5 |
| CHU Rouen - Hopital Charles Nicolle  1 rue de Germont  Dermatologie Rouen, 76031 | CPP SUD-EST II  Bâtiment Pinel  Groupement Hospitalier Est  59 Boulevard Pinel  Bron, 69500 |
| Hopital Larrey CHU de Toulouse  24 Chemin de Pouvourville Dermatologie  Toulouse, 31000 | CPP SUD-EST II  Bâtiment Pinel  Groupement Hospitalier Est  59 Boulevard Pinel  Bron, 69500 |
| HIA Sainte Anne  2 boulevard Sainte Anne  Dermatologie  Toulon, 83800 | CPP SUD-EST II  Bâtiment Pinel  Groupement Hospitalier Est  59 Boulevard Pinel  Bron, 69500 |
| Goethe Universität Frankfurt  Dept of Dermatology  Theodor Stern Kai 7, Haus 28  Frankfurt/ Main, Germany, 60590 | Ethikkommission bei der Ärztekammer Niedersachsen  Unterkommission zur Beurteilung medizinischer Forschung am Menschen  Karl-Wiechert-Allee 18-22  Hannover, 30625 |
| Fachklinik Bad Bentheim  Am Bade 1  Bad Bentheim  Niedersachsen, 48455 | Ethikkommission bei der Ärztekammer Niedersachsen  Unterkommission zur Beurteilung medizinischer Forschung am Menschen  Karl-Wiechert-Allee 18-22  Hannover, 30625 |
| Praxis Dr. med. Beate Schwarz - Germany  Bismarckstrasse 49  Langenau, 89129 | Landesärztekammer Baden-Württemberg Ethik-Kommission  Liebknechtstrasse 33  Stuttgart, 70565 |
| TFS Trial Form Support GmbH  Anckelmannsplatz 1  Hamburg, 20537 | Ethikkommission der Aerztekammer Hamburg  Humboldstrasse 67a  Ethikkommission der Ärztekammer Hamburg  Körperschaft des öffentlichen Rechts  Hamburg, 22083 |
| ISA - Interdisciplinary Study Association GmbH  Rankestrasse 34, 4th Floor  Berlin, 10789 | Ethikkommission bei der Ärztekammer Niedersachsen  Unterkommission zur Beurteilung medizinischer Forschung am Menschen  Karl-Wiechert-Allee 18-22  Hannover, 30625 |
| Hautarztpraxis  Am Bahnhof 1  Mahlow, 15831 | Ethik Kommission der Landesärztekammer Brandenburg - Hauptgeschäftsstelle  Dreifertstr 12  Cottbus, 03044 |
| Mensing Derma research GmbH  Heegbarg 4  Hamburg, 22391 | Ethikkommission der Aerztekammer Hamburg  Humboldstrasse 67a  Ethikkommission der Ärztekammer  Hamburg  Körperschaft des öffentlichen Rechts  Hamburg, 22083 |
| Medizinische Hochschule Hannover  Carl-Neuberg-Str. 1  Klinik fuer Dermatologie  Allergologie und Venerologie  Hannover, 30625 | Ethik-Kommission des Medizinischen Hochschule Hannover  Carl-Neuberg-Strasse 1  - OE 9151 -  Hannover, 30625 |
| Kume Clinic  1-65-2  Otorihigashimachi  Nishi Ku  Sakai City, Osaka Fu, 593-8324 | Sugiura Clinic IRB  4-4-16-301  Honcho  Kawaguchi-shi  Saitama, 332-0012 |
| Takagi Dermatology Clinic  Nishi-sanjo Minami 4-16  Dermatology  Obihiro-shi, Hokkaido, 080-0013 | Jimbo Orthopedics Institutional Review Board  5-38-41  Honcho, Koganei-city  Tokyo, 184-0004 |
| Sapporo Skin Clinic  2-1-1 Minami-3Jo Nishi  Chuo-ku  Sapporo, Hokkaido, 060-0063 | Sapporo Skin Clinic Institutional  Review Board  2-1-1 Minami-3Jo Nishi  Chuo-ku, Sapporo  Hokkaido, 060-0063 |
| Nzoz Przychodnia Specjalistyczna Medica  Waszyngtona 42 lok. 3  Czestochowa, 42-200 | Komisja Bioetyczna przy Dolnoslaskiej Izbie Lekarskiej we Wroclawiu  Kazimierza Wielkiego 45  Wroclaw, 50-077 |
| Klinika Ambroziak Estederm Sp. z o.o  Kosiarzy 9A  Warszawa, 02-953 | Komisja Bioetyczna przy Dolnoslaskiej Izbie Lekarskiej we Wroclawiu  Kazimierza Wielkiego 45  Wroclaw, 50-077 |
| Centrum Terapii Wspolczesnej J. M.  Jasnorzewska Spolka Komandytowo-Akcyjna  Kopcinskiego 21  Lodz, 90-242 | Komisja Bioetyczna przy Dolnoslaskiej Izbie Lekarskiej we Wroclawiu  Kazimierza Wielkiego 45  Wroclaw, 50-077 |
| Centrum Medyczne Matusiak w CITYCLINIC  Przychodnia Lekarsko-Psychologiczna Matusiak Spółka Partnerska  Sliczna 13  Wroclaw, 50566 | Komisja Bioetyczna przy Dolnoslaskiej Izbie Lekarskiej we Wroclawiu  Kazimierza Wielkiego 45  Wroclaw, 50-077 |
| DermoDent Centrum Medyczne Aldona Czajkowska Rafał Czajkowski s.c.  Tuberozy 3  Osielsko  86031 | Komisja Bioetyczna przy Dolnoslaskiej Izbie Lekarskiej we Wroclawiu  Kazimierza Wielkiego 45  Wroclaw, 50-077 |
| Royalderm Agnieszka Nawrocka  K.Kieślowskiego 3B/3  Warszawa, 02962 | Komisja Bioetyczna przy Dolnoslaskiej Izbie Lekarskiej we Wroclawiu  Kazimierza Wielkiego 45  Wroclaw, 50-077 |
| WroMedica I.Bielicka  A.Strzałkowska s.c.  ul. A. Mickiewicza 91  Wrocław, 51-685 | Komisja Bioetyczna przy Dolnoslaskiej Izbie Lekarskiej we Wroclawiu  Kazimierza Wielkiego 45  Wroclaw, 50-077 |
| Arlington Center for Dermatology  711 E Lamar Blvd Ste 200  Arlington, TX, 76011 | Sterling Institutional Review Board  6300 Powers Ferry Road, Suite 600-351  Atlanta, GA, 30339 |
| Virginia Clinical Research  6160 Kempville Road  Suite 200A  Norfolk, VA, 23502 | Sterling Institutional Review Board  6300 Powers Ferry Road, Suite 600-351  Atlanta, GA, 30339 |
| Austin Institute for Clinical Research  1601 E Pflugerville Pkwy, Suite 1101  Pflugerville, TX, 78660 | Sterling Institutional Review Board  6300 Powers Ferry Road, Suite 600-351  Atlanta, GA, 30339 |
| Clinical Partners  1524 Atwood Avenue  Suite 330  Johnston, RI, 02919 | Sterling Institutional Review Board  6300 Powers Ferry Road, Suite 600-351  Atlanta, GA, 30339 |
| Icahn School of Medicine at Mount Sinai  5 East 98th Street, 5th Floor  Dermatology  New York, NY, 10029 | Sterling Institutional Review Board  6300 Powers Ferry Road, Suite 600-351  Atlanta, GA, 30339 |
| Park Avenue Dermatology  906 Park Avenue  Orange Park, FL, 32073 | Sterling Institutional Review Board  6300 Powers Ferry Road, Suite 600-351  Atlanta, GA, 30339 |
| Psoriasis Treatment Center of Central New Jersey  59 One Mile Road  East Windsor, NJ, 08520 | Sterling Institutional Review Board  6300 Powers Ferry Road, Suite 600-351  Atlanta, GA, 30339 |
| Ohio State University  1328 Dublin Road  Columbus, OH, 43215 | WIRB  1019 39th Ave SE #120  Puyallup, WA, 98374 |
| Dawes Fretzin Clinical Research Group  7910 N Shadeland Avenue  Indianapolis, IN, 46256 | Sterling Institutional Review Board  6300 Powers Ferry Road, Suite 600-351  Atlanta, GA, 30339 |
| University of Pittsburgh Medical Center  Falk Medical Center  3601 Fifth Avenue, 5th Floor  Pittsburgh, PA, 15213 | Sterling Institutional Review Board  6300 Powers Ferry Road, Suite 600-351  Atlanta, GA, 30339 |
| Clinical NeuroScience Solutions Inc  6401 Poplar Avenue  Memphis, TN, 38119 | Sterling Institutional Review Board  6300 Powers Ferry Road, Suite 600-351  Atlanta, GA, 30339 |
| Progressive Clinical Research  1973 N.W. Loop 410, Suite 106  San Antonio, TX, 78213 | Sterling Institutional Review Board  6300 Powers Ferry Road, Suite 600-351  Atlanta, GA, 30339 |
| Arlington Dermatology  5301 Keystone Ct.  Rolling Meadows, IL, 60008 | Sterling Institutional Review Board  6300 Powers Ferry Road, Suite 600-351  Atlanta, GA, 30339 |
| Wolverine Clinical Trials  1200 N Tustin Ave, STE 240  Santa Ana, CA, 92705 | Sterling Institutional Review Board  6300 Powers Ferry Road, Suite 600-351  Atlanta, GA, 30339 |
| Oregon Dermatology and Research Center  2565 NW Lovejoy, Suite 200  Portland, OR, 97210 | Sterling Institutional Review Board  6300 Powers Ferry Road, Suite 600-351  Atlanta, GA, 30339 |
| California Allergy & Asthma Medical Group Inc.  1950 Sawtelle Blvd., Suite 138  Los Angeles, CA, 90025 | Sterling Institutional Review Board  6300 Powers Ferry Road, Suite 600-351  Atlanta, GA, 30339 |
| Indiana Clinical Trial Center  824 Edwards Drive, Suite 172  Plainfield, IN, 46168 | Sterling Institutional Review Board  6300 Powers Ferry Road, Suite 600-351  Atlanta, GA, 30339 |
| FORCARE CLINICAL RESEARCH, INC.  15416 North Florida Avenue  Tampa, FL, 33613 | Sterling Institutional Review Board  6300 Powers Ferry Road, Suite 600-351  Atlanta, GA, 30339 |
| Center for Clinical Studies  451 North Texas Avenue  Webster, TX, 77598 | Sterling Institutional Review Board  6300 Powers Ferry Road, Suite 600-351  Atlanta, GA, 30339 |
| Grekin Skin Institute  13450 E 12 Mile Rd  Warren, MI, 48088 | Sterling Institutional Review Board  6300 Powers Ferry Road, Suite 600-351  Atlanta, GA, 30339 |
| Premier Clinical Research  324 South Sherman, Suite A2  Spokane, WA, 99202 | Sterling Institutional Review Board  6300 Powers Ferry Road, Suite 600-351  Atlanta, GA, 30339 |

**LUNA (NCT04990440)**

| **Site** | **Institutional Review Board/Ethical Committee** |
| --- | --- |
| CARE - Centro de Alergia y Enfermedades Respiratorias  Luis Maria Drago 250  Ciudad Autonoma de Buenos Aires 1414 | Comité de Etica en Investigación Clínica (CEIC)  Larrea 1381 3°"A" Caba, C1117ABK |
| Clínica Adventista Belgrano Estomba 1710  CABA  Buenos Aires C1430EGF | Comité de Etica en Investigación Clínica (CEIC)  Larrea 1381 3°"A" Caba, C1117ABK |
| STAT Research S.A.  Av. Callao 875 3°E and F CABA  C1023AAB | Comité de Etica en Investigación Clínica (CEIC)  Larrea 1381 3°"A" Caba, C1117ABK |
| Conexa Investigacion Clinica S.A. Libertad 1213 2nd floor  CABA  Buenos Aires C1015AAA | Comité de Etica en Investigación Clínica (CEIC)  Larrea 1381 3°"A" Caba, C1117ABK |
| CINME - Centro de Investigaciones Metabolicas  Uriburu 774 3rd Floor  Unique building  Ciudad de Buenos Aires C1027AAP | Comité de Ética en Investigación (CINME)  Viamonte 2278 Buenos Aires C1056ABJ |
| Vital Prospects Clinical Research Institute PC  7307 S. Yale Avenue Suite 200  Tulsa, OK 74136 | Sterling IRB  6300 Powers Ferry Road, Suite 600-351  Atlanta, GA 30339 |
| Modern Research Associates  9101 N. Central Expressway, Suite 170  Dallas, TX 75231 | Sterling IRB  6300 Powers Ferry Road, Suite 600-351  Atlanta, GA 30339 |
| Texas Dermatology and Laser Specialists  3320 Oakwell Court  San Antonio, TX 78218 | Sterling IRB  6300 Powers Ferry Road, Suite 600-351  Atlanta, GA 30339 |
| Dawes Fretzin Clinical Research Group  7910 N Shadeland Avenue  Indianapolis, IN 46256 | Sterling IRB  6300 Powers Ferry Road, Suite 600-351  Atlanta, GA 30339 |
| Progressive Clinical Research  1973 N.W. Loop 410, Suite 106  San Antonio, TX 78213 | Sterling IRB  6300 Powers Ferry Road, Suite 600-351  Atlanta, GA 30339 |
| Clinical Research Institute of Michigan, LLC  30795 23 Mile Rd, Ste 206  Chesterfield, MI 48047 | Sterling IRB  6300 Powers Ferry Road, Suite 600-351  Atlanta, GA 30339 |

# Supplementary methods

Study 1 was conducted by XBiotech between 07May2018 and 11Dec2018, at 9 centers in the US. Study 2 was conducted by XBiotech between 29Oct2019 and 17Nov2020, at 15 sites in the US. After acquiring bermekimab from XBiotech on 07Dec2019, Janssen Research & Development, LLC, conducted the GENESIS study between 17May2021 and 31Mar2022 (early study termination date) at 45 centers that enrolled patients in Canada, Germany, Japan, Poland, and the US, and the LUNA study was conducted between 21Aug2021 and 02Feb2022 (early study termination date) at 4 centers that enrolled patients in the US and Argentina.

## Study designs

The phase 2 studies used prefilled syringes containing a sterile liquid formulation of bermekimab for a 2.0 mL injection in a stabilizing isotonic formulation buffer at pH 6.2-6.5, containing non-active ingredients of low concentrations of sodium phosphate, citric acid, and trehalose, at varying concentrations.

***Study 1***

This open‑label, dose escalation, phase 2 study (NCT03496974) was conducted in 2 separate cohorts of patients with atopic dermatitis (AD). Following an initial screening visit (week 0), 10 patients in group A received every week (qw) bermekimab 200 mg doses via subcutaneous (SC) administration for 3 weeks (total of 4 doses), with 2 follow-up visits. Dose escalation to group B, where 28 patients received 400 mg doses SC qw for 7 weeks (8 doses total), was initiated after confirmation that no safety signals were identified and no study stopping rules, including death or ≥2 cumulative SAEs, were met in group A.

***Study 2***

This randomized, double-blind, placebo-controlled, phase 2 study (NCT04021862) evaluated SC bermekimab in patients with AD. The planned total sample size was approximately 90 patients (ie, 30 patients per treatment arm). Using an electronic randomization system, patients were randomized in a 1:1:1 allocation ratio to 1 of 3 treatment arms: SC bermekimab 400 mg at week 0 and then qw for weeks 1 to 31; SC bermekimab 800 bermekimab at week 0 (loading dose) followed by bermekimab 400 mg q2w for weeks 1 to 31; placebo qw from weeks 1 to 15, then SC bermekimab 400 mg qw for weeks 16 to 31 (crossover period). The randomization schedule was stratified by baseline Eczema Area and Severity Index (EASI) severity (less severe [EASI≥16 and<28] or more severe [EASI≥28]). Individual study duration was approximately 40 weeks (30-day screening period, 32-week treatment period, and a 4-week follow-up period). Patients were required to apply moisturizers at least twice daily before randomization and to continue use of moisturizers throughout the study. In order to maintain blinding, all patients received 2 SC injections at week 0, followed by injections qw from week 1 to 31. A qualified individual was designated at each study site to perform all preparation and injection of the investigational product. The designated injector was documented in the source documents at each visit and could have no other contact with the patient during the study. The Investigator was not provided with randomization codes. All site personnel and patients remained blinded to the treatment assignments until the final patient had completed the week 36 evaluations, and the database had been locked.

Concomitant medications, such as antihistamines and/or nonsteroidal anti-inflammatory drugs (ie, ibuprofen), were allowed, based on the Investigator’s judgment, in order to prevent or treat injection site reaction or discomfort, and ≤2 bleach baths were permitted per week. During the study, systemic and topical corticosteroids were prohibited but were allowed as rescue medication.

One amendment to the protocol occurred on 22Jan2020 with changes that included removal of open-label period, extension of the study period to approximately 40 weeks (including screening, treatment period, and follow-up), revision of the rescue treatment section to allow for alternative treatment options without discontinuation, and additional visits at which the patients needed to be monitored for a minimum of 30 minutes after study agent administration.

***GENESIS***

This randomized, double-blind, placebo- and active-comparator-controlled, multicenter, interventional phase 2b study (NCT04791319) (**Fig E3**) had a planned sample size of approximately 200 patients. Patients were randomly assigned in a 1:1:2:2 ratio to 1 of 4 treatment groups: placebo; bermekimab 350 mg SC qw; bermekimab 700 mg SC qw; comparator/reference arm (dupilumab 600 mg at week 0 followed by dupilumab 300 mg q2w from weeks 2 through 14). The less viscous bermekimab dose of 350 mg qw was selected for this study instead of 400 mg used in previous studies. A sterile liquid formulation of bermekimab 350 mg (175 mg/mL) with an injectable volume of 2.0 mL was used. Commercially available dupilumab was provided by the sponsor as a sterile liquid formulation of 150 mg/mL with an injectable volume of 2.0 mL. Patients were required to apply moisturizers at least once a day for ≥7 days before randomization and continue use of moisturizers throughout the study.

At week 16, patients who achieved 75% improvement in the EASI (EASI-75) response were to be rerandomized in a 1:1 ratio, either to continue to receive bermekimab 700 mg qw or to receive bermekimab 350 mg qw, through week 31; patients who did not achieve an EASI-75 response were to continue to receive bermekimab 700 qw through week 31. Patients who achieved an EASI-75 response at week 16 were to continue on dupilumab 300 mg q2w from week 16 through week 30; dupilumab nonresponders were to receive placebo qw at weeks 16 through 18 and bermekimab 700 mg qw from weeks 19 through 31.

Two planned database locks were to occur after all patients had completed the week 16 and week 36 (end of study) visits. An interim analysis was planned when approximately 50% of patients had completed the week 16 visit. An independent, internal committee was planned to review the results of the interim analysis, and an independent, external data monitoring committee reviewed unblinded safety data to ensure the safety of the patients enrolled in the study.

The protocol was amended on 02Jun2021 with clarifications to provide guidance regarding the use of rescue therapy; correction in calculation error in total volume of blood sample to be collected; whether the Janus kinase inhibitors listed as prohibited treatments referred to systemic and/or external preparations, and inclusion of additional safety measures related to 12-lead electrocardiogram.

***LUNA***

This double-blind, randomized, placebo-controlled, multicenter, interventional phase 2 study (NCT04990440) (**Fig E4**) had 3 periods planned: screening for up to 4 weeks, a double-blind placebo-controlled period of 16 weeks, and a safety follow-up period of 4 weeks, including an end of study visit.

Patients were to be randomized in a 4:1 ratio to receive an intravenous (IV) infusion of either bermekimab or placebo. Approximately 10 patients were planned to receive bermekimab 800 mg IV qw or placebo in Part A; Part B was to consist of approximately 30 patients receiving bermekimab 1200 mg IV qw or placebo and Part C was planned to consist of approximately 20 patients receiving bermekimab or placebo at a dose of ≥800 mg and ≤2400 mg IV qw. A sterile liquid formulation of 350 mg (175 mg/mL) bermekimab was provided in a prefilled syringe with an injectable volume of 2.0 mL. Patients were required to use moisturizers at least once a day for ≥7 days before randomization and to continue throughout the study.

One protocol amendment occurred on 23Nov2021 and included additional sample collections for lipid panels to provide a more robust safety check for patients.

## Patients

All patients provided written informed consent to participate in the study, and all four phase 2 studies included patients who were at least 18 years old who had an inadequate response to topical treatment for AD, failed to tolerate topical medications or topical medications were medically inadvisable, and had chronic AD for ≥3 years (Study 1 and Study 2) or for ≥1 year (GENESIS and LUNA). Patients had an EASI score ≥16, an IGA score ≥3, and ≥10% body surface involvement at both screening and baseline. Study 2 also included patients with baseline pruritis NRS scores for maximum intensity ≥3, based on the average of daily pruritis NRS scores for maximum itch intensity reported during the 7 days prior to randomization. Patients were excluded if they had skin comorbidities that may interfere with study assessments; if they had open, infected skin wounds or ulcers or superficial skin infections within 1 week of baseline visit; and due to prior use of topical corticosteroids or topical calcineurin inhibitors within 7 to 28 days prior to baseline to treat AD or use of prescription moisturizers or moisturizers containing additives initiated during the screening period.

Specific concomitant medications/procedures that were prohibited prior to or during the studies included: **Study 1—**topical corticosteroids or topical calcineurin inhibitors within 1 week before baseline visit; phototherapy for AD or immunosuppressive/immunomodulating drugs within 4 weeks of baseline; and regular use (≥2 visits per week) of tanning booth within 4 weeks of screening visit; **Study 2—**topical tacrolimus and pimecrolimus, topical corticosteroids, topical calcineurin inhibitors, systemic corticosteroids (except if required for AD rescue), leukotriene inhibitors, allergen immunotherapy; investigational AD drug within 30 days or 5 half-lives of baseline; immunosuppressive/immunomodulatory drugs or phototherapy within 4 weeks of baseline; concomitant ultraviolet procedures; and tanning in a bed/booth; **GENESIS—**initiation of treatment of AD with any prescription topicals including moisturizers during the screening period, or use of any prescription topical treatment for AD within 1 week of the baseline visit; having previously received dupilumab or any IL-1 antagonist; receiving systemic immunosuppressants or phototherapy or any systemic medications/treatments that could affect AD or IGA evaluations within 4 weeks of the first administration of any study intervention; immunomodulating biologic therapy within 3 months; natalizumab, belimumab, or agents that modulate B or T cells within 12 months; **LUNA**—dupilumab or any IL-1 antagonist; immunomodulating biologic therapy within 3 months of baseline; systemic immunosuppressant, immunomodulatory, or cytotoxic treatments within 4 weeks of baseline; natalizumab, belimumab, or agents that modulate B or T cells within 12 months of baseline; investigational AD drug within 30 days or 5 half-lives of baseline; topical corticosteroids (except for rescue therapy); topical calcineurin inhibitors; any prescription topical treatment for AD; oral systemic corticosteroids (except for a maximum of 10 days for standard care for conditions other than AD); phototherapy within 4 weeks of baseline; and bleach baths.

## Study objectives and endpoints

***Study 1***

The primary endpoints were safety and tolerability. Adverse events were classified according to the National Cancer Institute Common Terminology Criteria for AEs. Key secondary endpoints evaluated characteristics of the patients’ AD with changes in EASI, IGA response, NRS worst itch, and NRS pain. Other secondary endpoints included Dermatology Life Quality Index, Patient Oriented Eczema Measure, Hospital Anxiety Depression Scale, and Severity Scoring of AD score.

***Study 2***

The primary objectives were to evaluate efficacy by assessing the percentage of patients achieving EASI-75 at week 16 and to assess the safety and tolerability of bermekimab. Secondary objectives included further evaluation of bermekimab efficacy by assessing reduction in signs and symptoms of AD, effect on dermatologic health-related quality of life, psychological disease impact, and evaluation of pharmacokinetics and immunogenicity.

***GENESIS***

The primary endpoint of this study was to evaluate efficacy using EASI-75 at week 16. The secondary objectives included additional efficacy assessments at week 16: proportion of patients with both validated IGA-AD of 0 or 1 and a reduction from baseline of ≥2 points; proportion of patients with improvement (reduction) of eczema-related itch NRS ≥4 from baseline to week 16 among patients with baseline itch value ≥4; proportion of patients with EASI-90. In addition, the efficacy of bermekimab was evaluated at week 16 relative to dupilumab with respect to the proportion of patients with EASI-75, the proportion of patients with EASI-90, and the proportion of patients with both validated IGA-AD of 0 or 1 (5‑point scale) and a reduction from baseline of ≥2 points. The safety and tolerability, including AEs, pharmacokinetics, and immunogenicity were also evaluated.

***LUNA***

The primary objective was to evaluate the efficacy of 16 weeks of multiple IV doses of bermekimab compared to placebo using the proportion of patients with EASI-75 at week 16. Secondary objectives were to evaluate the PK, immunogenicity, and the safety and tolerability of 16 weeks of multiple IV doses of bermekimab, including AEs, SAEs, infections, changes in laboratory assessments, and abnormal vital signs.

## Statistical analysis

Treatment-emergent AEs, hereafter referred to as AEs, were coded in accordance with the Medical Dictionary for Regulatory Activities (MedDRA) – Study 1, Version 21.0; Study 2, Version 23.0; GENESIS, Version 24.1; and LUNA, Version 23.0 – and the system order class.

**Study 1**. A non-probability convenience sampling method was used for obtaining the basic data and trends on efficacy measures. Although 20 patients were initially planned to be enrolled, this was expanded to allow for 38 total patients (Group A=10, Group B=28). Patients who received rescue treatment during the study were considered treatment failures but continued study treatment if rescue consisted of topical medications. Missing data were not categorized in the summaries. If baseline assessment was not available, the patient was not included in baseline summaries. No adjustment for multiple comparisons was made. All primary and secondary efficacy analyses were performed for the safety analysis set, which consisted of all patients who received ≥1 dose of study drug, and the protocol population, which consisted of patients who completed both baseline and visit 8 assessments. Corresponding confidence interval of the point estimate of mean change was provided by treatment group and for overall pooled population. Summary statistics on Dermatology Life Quality Index, EASI, Hospital Anxiety Depression Scale (for anxiety and depression), Patient Oriented Eczema Measure, Severity Scoring of AD, and weekly average NRS pruritus (worst itch and average itch) and pain NRS scores were generated using individual patient level data on respective visits.

**Study 2**. The full analysis set was used for the efficacy analyses of the endpoints, unless otherwise specified. The sample size was calculated for comparison of the bermekimab 400 mg qw injection group with the placebo group. Assuming EASI-75 would be achieved in 60% of the intent-to-treat patients treated in the bermekimab 400 mg qw injection group compared with 20% in placebo-treated patients, then for a randomization ratio of 1:1:1, a sample size of 69 patients (23 in each treatment arm) was required for ~80% power at a 2‑sided 0.05 significance level using a Pearson chi-square test.

**GENESIS*.*** Approximately 200 patients were planned to be randomized in a 1:1:2:2 ratio to the placebo (n=33), bermekimab 350 mg (n=33), bermekimab 700 mg (n=67), or dupilumab (n=67) treatment groups. These sample sizes provided the study with ≥88% power to detect a treatment difference between bermekimab treatment groups and placebo group in EASI-75 at week 16, based on a 2-sample Z-test at a Type I error rate of 0.05 (2-sided), and also detected a treatment difference between dupilumab and placebo in EASI-75 at week 16 at a 2-sided significance level of 0.05.

**LUNA.** The planned enrollment of approximately 60 patients was to provide sufficient power to detect a difference between the patients receiving bermekimab and the patients receiving placebo for the primary endpoint of the proportion of patients achieving EASI-75 at week 16. Based on the assumption that the EASI-75 response at week 16 would be 15% for placebo and 65% to 70% for the bermekimab treatment population, approximately 60 patients (10 in Part A, 30 in Part B, and 20 in Part C) were planned to be randomized. In each part, patients were to be randomized in a 4:1 ratio to bermekimab or placebo. Thus, there would be 8 patients in the bermekimab 800 mg group, 24 patients in the bermekimab 1200 mg group, 16 patients in the bermekimab Part C dose, and 12 patients total across the placebo groups. These sample sizes provide the study with ≥80% power to detect a treatment difference between each bermekimab intervention group and the pooled placebo intervention group in EASI-75 at week 16, based on a Fisher’s Exact test at a Type I error rate of 0.1 (2-sided).

# Supplementary table

## Table S1 Study 1 adverse events

|  | Number (%) of Patients | | | | | | | |
| --- | --- | --- | --- | --- | --- | --- | --- | --- |
|  | **200 mg Bermekimab qw Group (N=10)** | | | | **400 mg qw Bermekimab Group (N=28)** | | | |
| AE MedDRA Preferred Term | **Grade 1** | **Grade 2** | **Grade 3** | **Total** | **Grade 1** | **Grade 2** | **Grade 3** | **Total** |
| Lymphadenopathy | 0 | 0 | 0 | 0 | 1 (3.6) | 0 | 0 | 1 (3.6) |
| Sinus tachycardia | 1 (10) | 0 | 0 | 1 (10) | 0 | 0 | 0 | 0 |
| Abdominal pain | 0 | 0 | 0 | 0 | 1 (3.6) | 0 | 0 | 1 (3.6) |
| Nausea | 0 | 0 | 0 | 0 | 2 (7.1) | 0 | 0 | 2 (7.1) |
| Vomiting | 0 | 0 | 0 | 0 | 1 (3.6) | 0 | 0 | 1 (3.6) |
| Injection site erythema | 1 (10) | 0 | 0 | 1 (10) | 2 (7.1) | 0 | 0 | 2 (7.1) |
| Oedema peripheral | 0 | 0 | 0 | 0 | 0 | 1 (3.6) | 0 | 1 (3.6) |
| Peripheral swelling | 0 | 0 | 0 | 0 | 1 (3.6) | 0 | 0 | 1 (3.6) |
| Urinary tract infection | 0 | 0 | 0 | 0 | 1 (3.6) | 0 | 0 | 1 (3.6) |
| Wheezing | 0 | 0 | 0 | 0 | 1 (3.6) | 1 (3.6) | 0 | 2 (7.1) |
| Swelling face | 0 | 0 | 0 | 0 | 1 (3.6) | 0 | 0 | 1 (3.6) |
| Diastolic hypertension | 0 | 1 (10) | 0 | 1 (10) | 0 | 0 | 0 | 0 |
| Hypertension | 0 | 0 | 1 (10) | 1 (10) | 0 | 0 | 0 | 0 |
| Systolic hypertension | 0 | 1 (10) | 0 | 1 (10) | 0 | 0 | 0 | 0 |

AE, adverse event; MedDRA, Medical Dictionary for Regulatory Activities; N, number of patients.

# Supplementary figures

## **Fig. S1** Study 1 (open-label) patient flow diagram and disposition

**
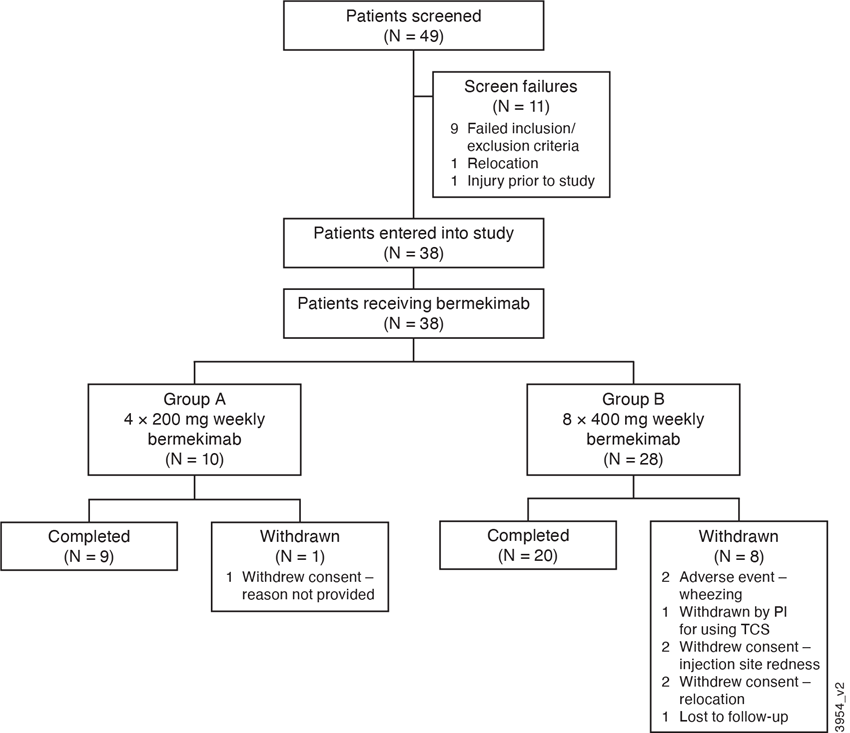
**

N, number of patients; PI, Principal Investigator; TCS, topical corticosteroids.

## **Fig. S2** Study 2 design (double-blinded, randomized, placebo-controlled) and patient disposition

**
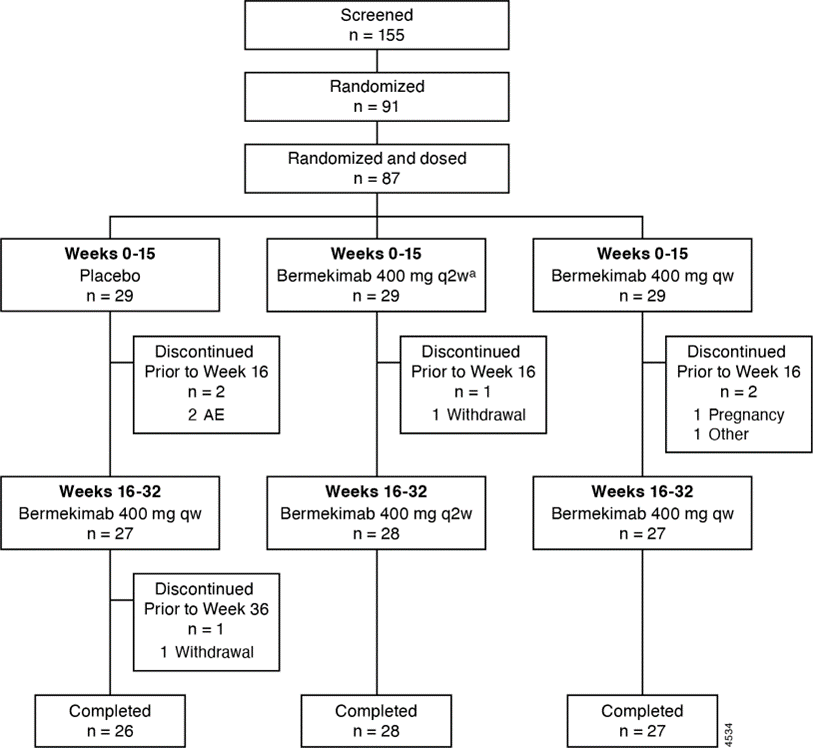
**

^a^With a loading dose of 800 mg bermekimab at week 0. qw, every week; q2w. every 2 weeks.

## **Fig. S3** GENESIS study design (double-blind, randomized, placebo-controlled, comparator-controlled) and patient disposition


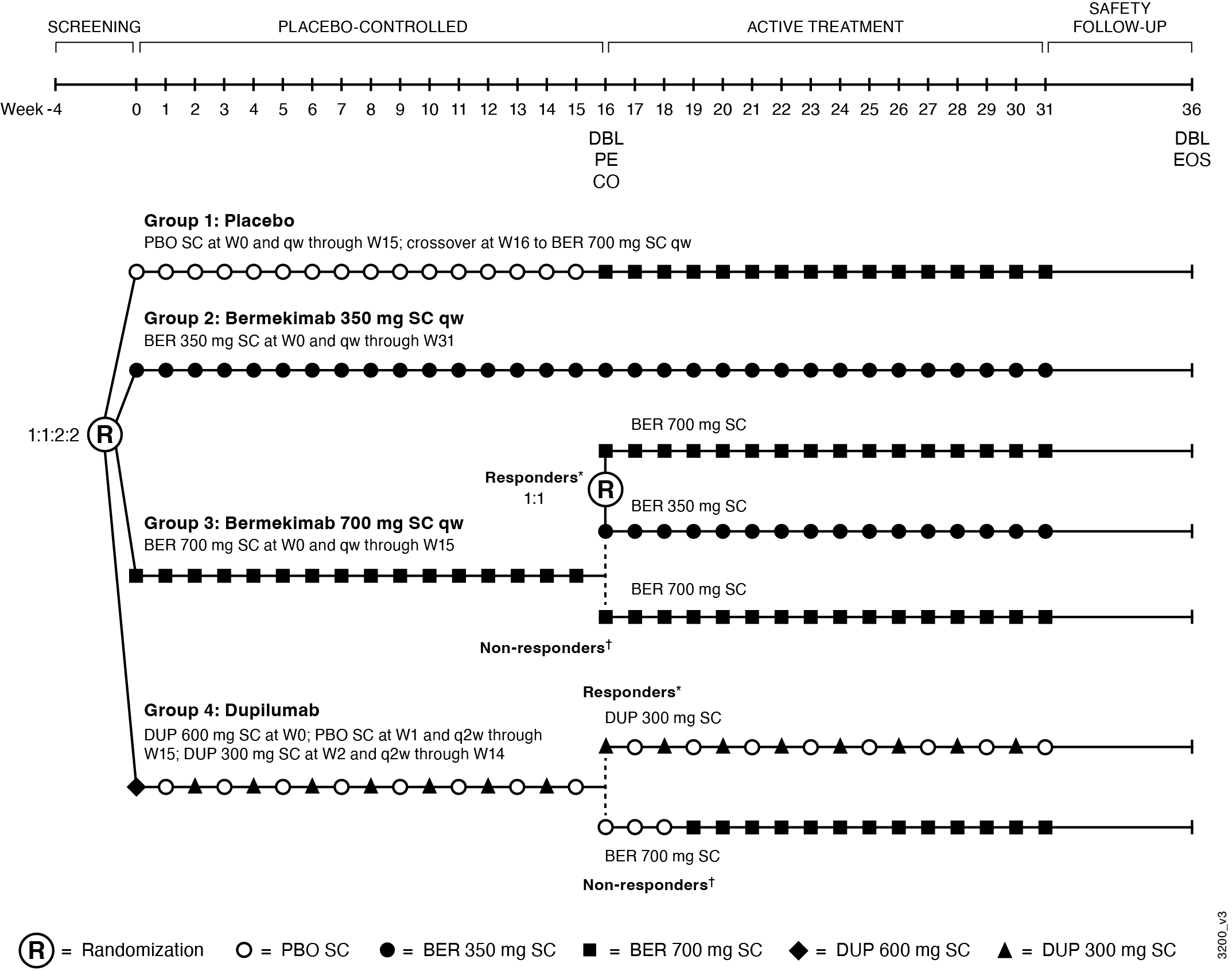


^*^Patients who achieve a 75% improvement of Eczema Area and Severity Index from baseline (EASI-75) response at week 16. †Patients who do not achieve an EASI‑75 response at week 16. BER, bermekimab; CO, crossover; DBL, database lock; DUP, dupilumab; EOS, end of study; PBO, placebo; PE, primary endpoint; qw, every week; q2w, every 2 weeks; SC, subcutaneous; W, week.

## **Fig. S4** LUNA study design


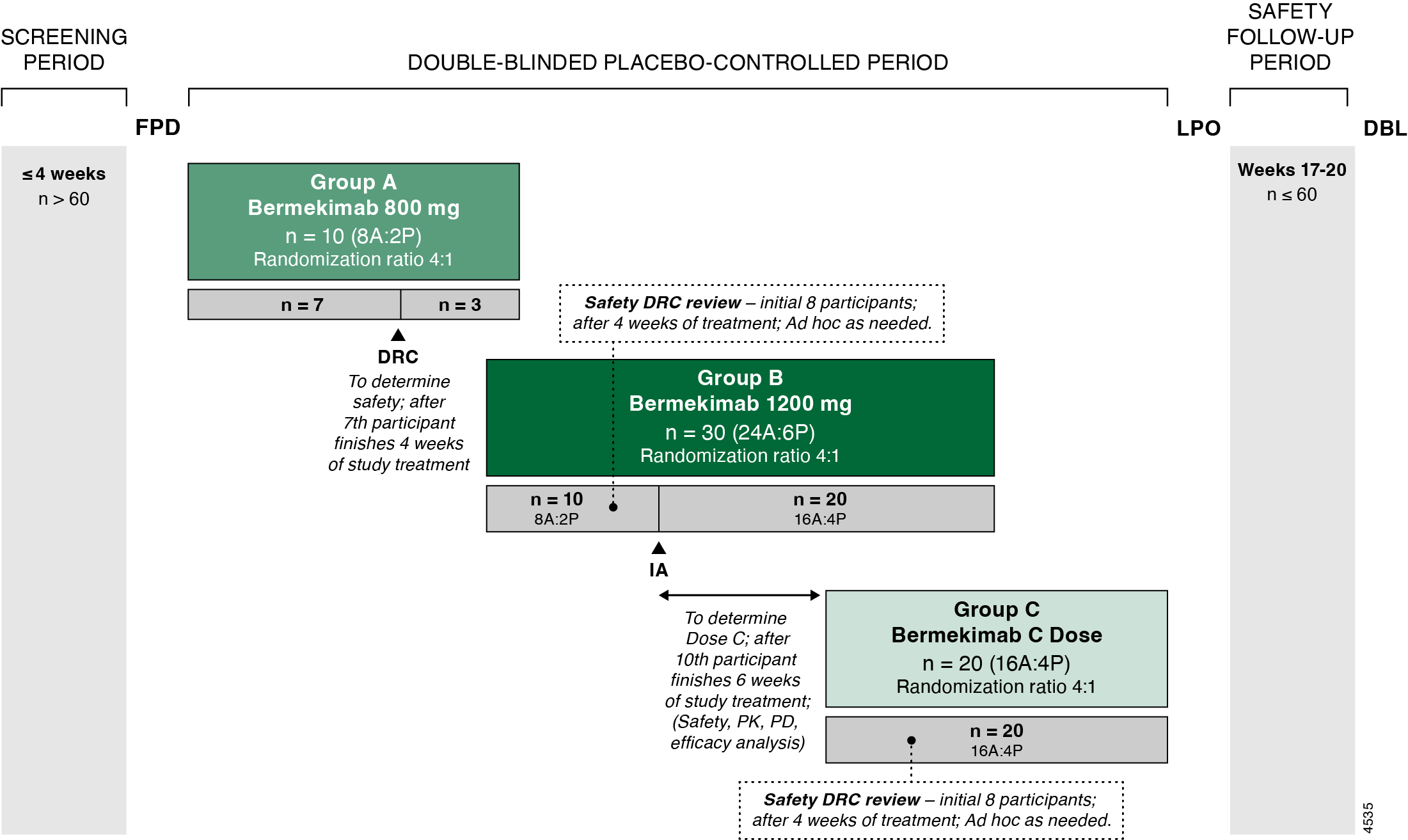


The DRC in Part A was planned to review unblinded safety data after the 7th patient had completed week 4 dose administration, and it was planned to include the first 7 patients in Part A. The DRC in Part B was planned to review unblinded safety data after the 8th patient had completed week 4 dose administration, and it was planned to include the first 8 patients in Part B. The DRC in Part C was planned to review unblinded safety data after the 8th patient had completed week 4 of dose administration, and it was planned to include the first 8 patients in Part C. Ad hoc DRC was also planned to convene if requested by the clinical team. A, administered bermekimab; DBL, database lock; DRC, Data Review Committee; FPD, first patient dosed; IA, Interim Analysis; LPO, last patient out; P, placebo; PD, pharmacodynamics; PK, pharmacokinetics.

## **Fig. S5** Design of the novel phase 0 (NCT03953196) ex vivo pharmacodynamic skin assay


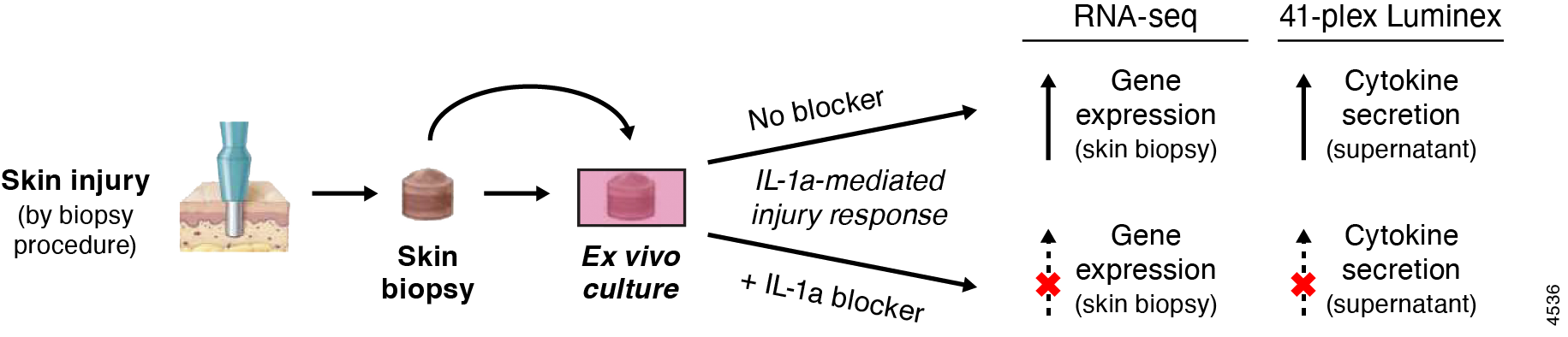


This assay was developed to monitor proteomic and transcriptomic changes associated with blockade of the IL-1α-mediated, injury-induced, inflammatory response and to further support the understanding of the pharmacokinetics/pharmacodynamics of bermekimab. Skin biopsies (4 mm) from healthy subjects were cultured ex vivo for 30 seconds (control) or 24 hours to allow for the induction of an IL-1α-mediated injury response in the presence or absence of an anti-IL1α monoclonal antibody (R&D Systems, clone No. 4414). This assay demonstrated that IL-1α is upregulated in an ex vivo culture of skin biopsies from healthy donors at 24 hours compared to control. Ex vivo IL-1α blockade resulted in a reduction in protein levels of inflammatory mediators and injury-induce gene expression (data not shown). IL‑1a, interleukin-1α; RNA-seq, RNA-sequencing.

## **Fig. S6** Implementation of the ex vivo pharmacodynamic skin assay in a bermekimab phase 1 study (NCT04544813)


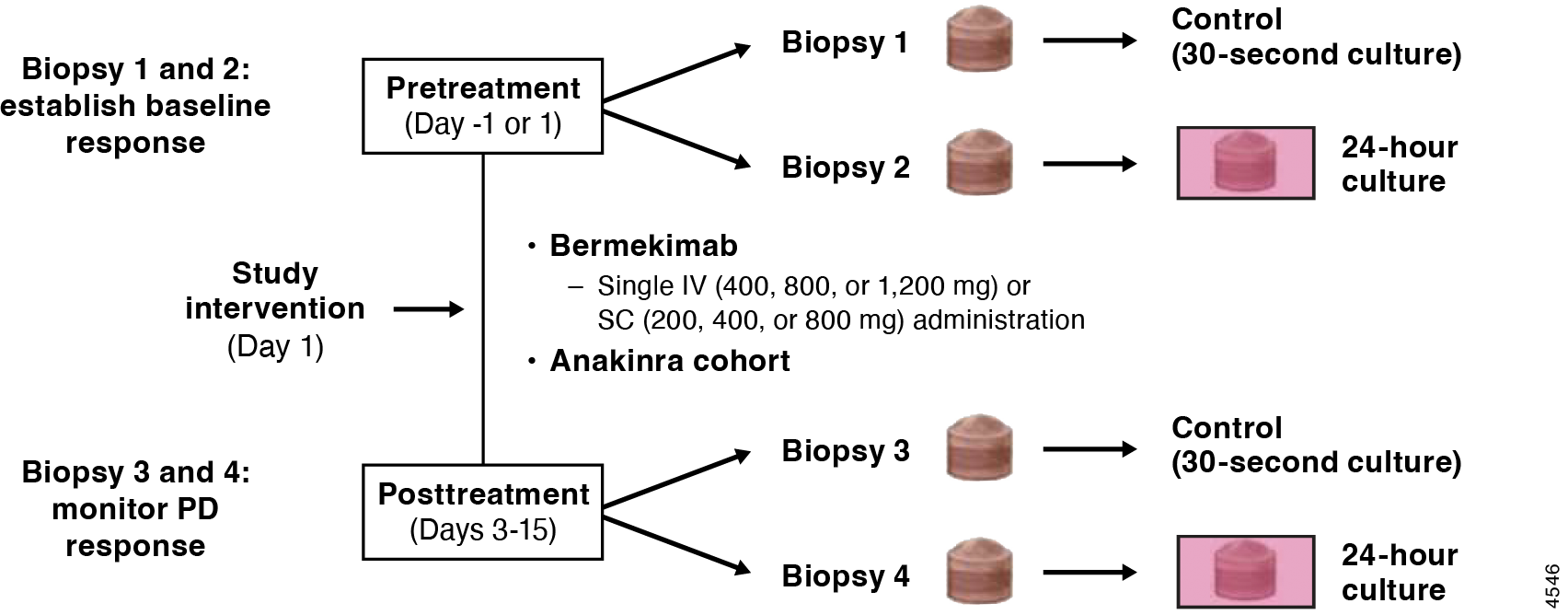


Two skin biopsies at each time point were obtained from healthy volunteers (n=78) at baseline and followed by a single IV dose of bermekimab (400, 800, or 1200 mg). Skin biopsies were collected before and after bermekimab administration from each subject in order to monitor PD effects in the ex vivo skin assay, with SC doses of bermekimab (200, 400, or 800 mg), or a 3-day SC injection of anakinra, an IL-1 receptor antagonist (100 mg). Pretreatment biopsies were used to establish baseline response, while post-treatment biopsies were used to monitor the bermekimab PD response. One of 2 pretreatment and post-treatment biopsies were cultured for 24 hours to allow for induction of an IL-1α-mediated inflammatory response, while the other 2 biopsies served as controls (30-second culture). Inflammatory cytokine and chemokine proteins were upregulated with 24 hours of ex vivo culture compared to control before treatment with bermekimab 800 mg SC. Ex vivo culture supernatant from patients treated with bermekimab (1200 mg IV) demonstrated lower proinflammatory cytokine and chemokine protein expression. Inflammatory cytokine and chemokine proteins were downregulated in post-bermekimab treatment (200, 400, or 800 mg SC) skin explants compared to pre-bermekimab treatment skin explants. Bermekimab induced a dose-dependent change in 73 genes from pre- to post-treatment with the IL-1 receptor antagonist anakinra only showed modest effect. IL, interleukin; IV, intravenous; PD, pharmacodynamic; SC, subcutaneous.

## **Fig.** **S7** Heatmap of proteins downregulated in culture supernatant of 200, 400, and 800 mg SC bermekimab-treated skin explants compared to pretreatment in the phase 1 bermekimab pharmacodynamic assay, showing levels of analytes in ex vivo culture supernatant pre- and post-treatment with SC bermekimab (200 mg, 400 mg, or 800 mg)


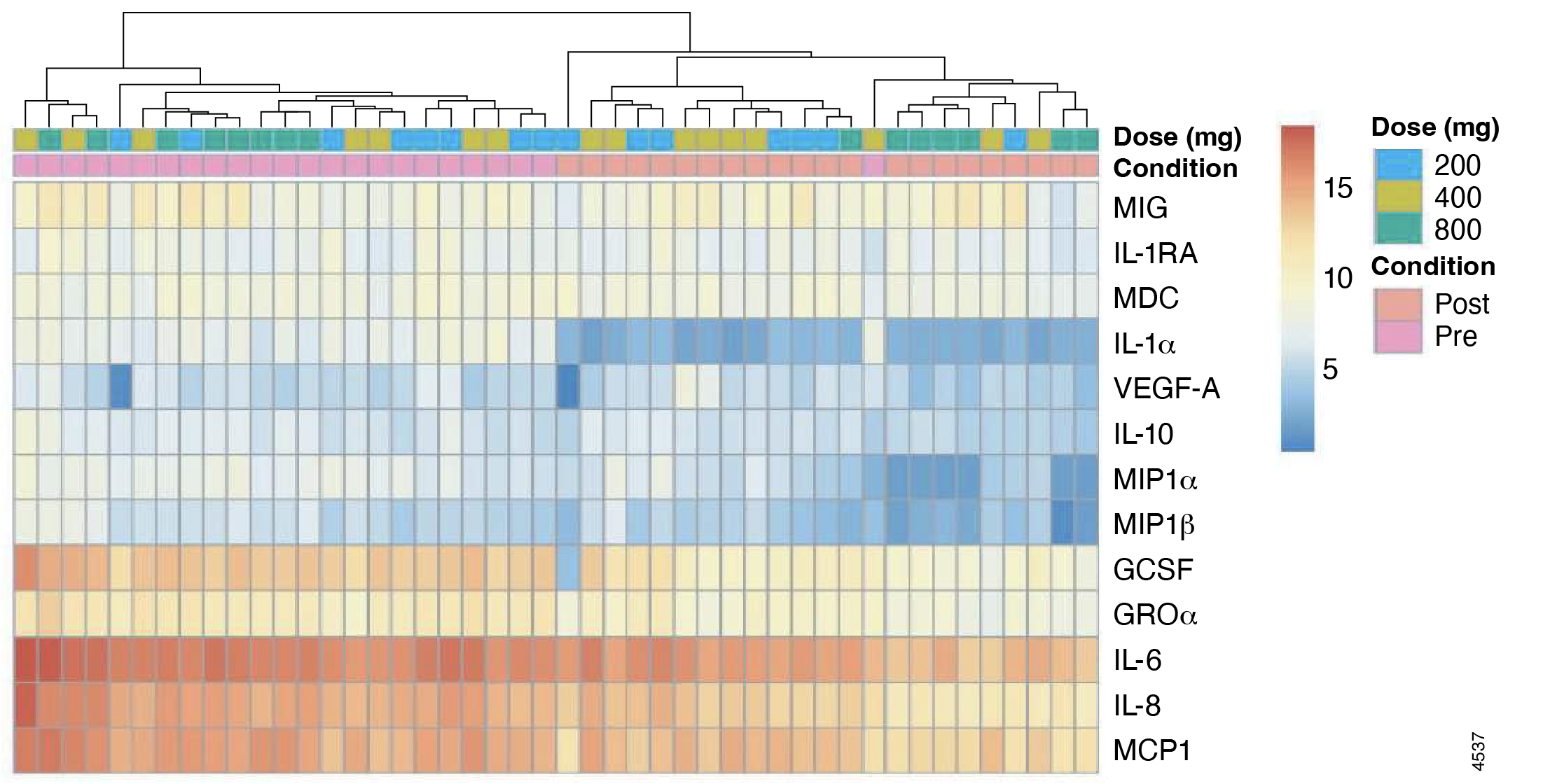


Proteins that were upregulated with a geometric mean induction fold change >5 in all 3 dosing cohorts are included in the heatmap. Scale denotes log2 of analyte concentration measured in assay (pg/mL). Inflammatory cytokine and chemokine proteins were downregulated in post-bermekimab-treated (200, 400, or 800 mg SC) skin explants compared to pre-bermekimab treatment of skin explants. GCSF, granulocyte colony-stimulating factor; GRO, growth-regulated oncogene (CXC motif chemokine ligand 1); IL, interleukin; IL-1RA, interleukin 1 receptor antagonist; MCP, monocytic chemoattractant protein; MDC, macrophage-derived chemokine; MIG, monokine induced by interferon-γ (CSC motif chemokine ligand 9); MIP, macrophage inflammatory protein; SC, subcutaneous; VEGF-A, vascular endothelial growth factor A.

## **Fig. S8** Dose-dependent reduction of protein expression from ex vivo culture supernatant in pretreatment versus post-treatment SC bermekimab administration conditions


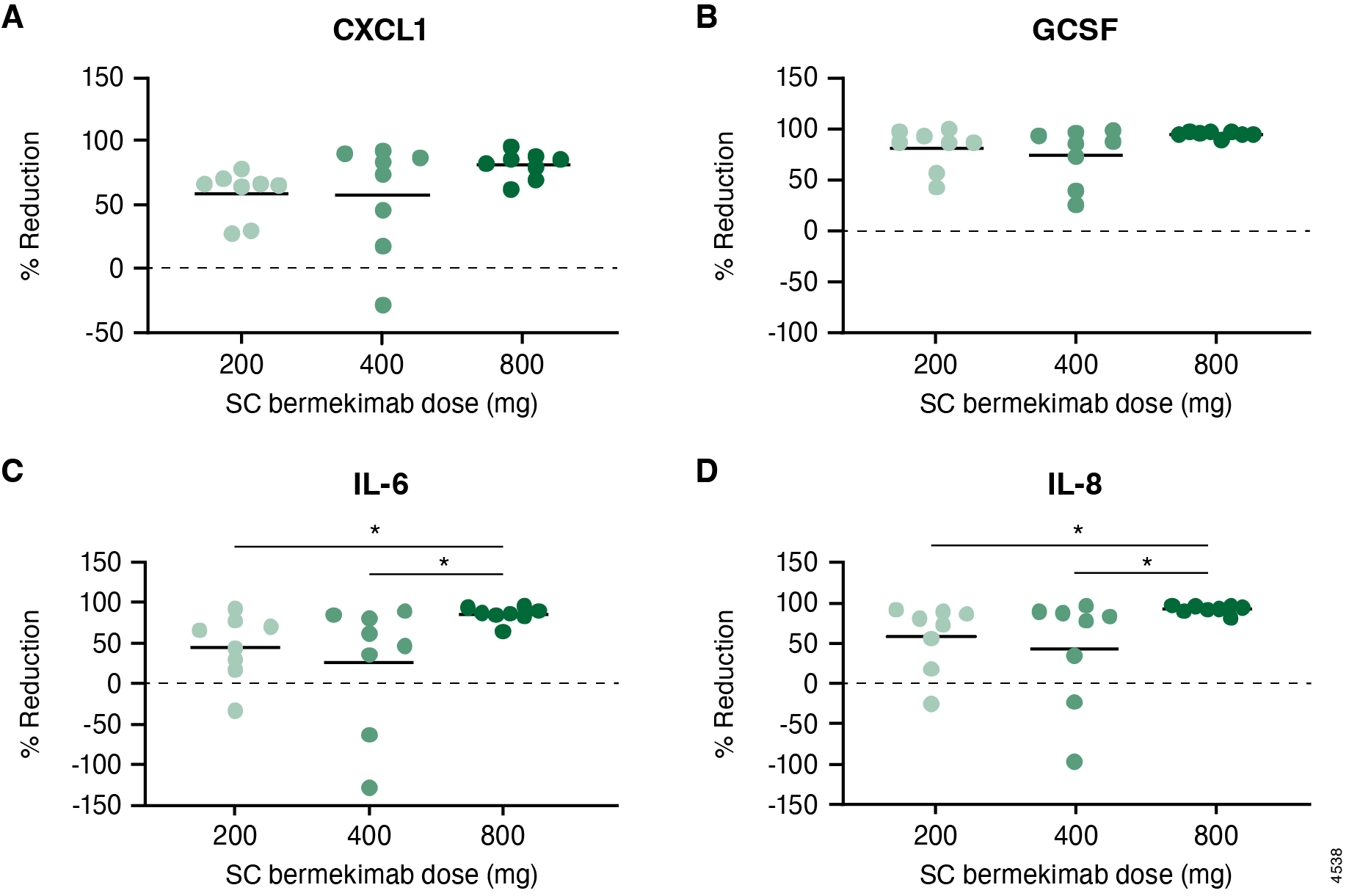


Percent reduction in CXCL1 (**a**), GCSF (**b**), IL-6 (**c**), and IL-8 (**d**) analytes in ex vivo culture supernatant samples from 200 mg, 400 mg, and 800 mg bermekimab SC cohorts. Statistical analysis was performed using Kruskal-Wallis test (**P*<0.05). CXCL1, CXC motif chemokine ligand; GCSF, granulocyte colony-stimulating factor; IL, interleukin; SC, subcutaneous.
